# VGG‐EffAttnNet: Hybrid Deep Learning Model for Automated Chili Plant Disease Classification Using VGG16 and EfficientNetB0 With Attention Mechanism

**DOI:** 10.1002/fsn3.70653

**Published:** 2025-07-24

**Authors:** Ritu Rani, Salil Bharany, Dalia H. Elkamchouchi, Ateeq Ur Rehman, Rahul Singh, Seada Hussen

**Affiliations:** ^1^ Chitkara University Institute of Engineering and Technology Chitkara University Rajpura Punjab India; ^2^ Department of Information Technology, College of Computer and Information Sciences Princess Nourah bint Abdulrahman University Saudi Arabia; ^3^ School of Computing Gachon University Republic of Korea; ^4^ Department of Electrical Power Adama Science and Technology University Adama Ethiopia

**Keywords:** attention mechanism, automated detection, chili plant disease, deep learning, EfficientNet, hybrid model, VGG16

## Abstract

Chili plant diseases significantly impact global agriculture, necessitating accurate and rapid classification for effective management. The study introduces VGG‐EffAttnNet, a hybrid deep learning model combining VGG16 and EfficientNetB0 with attention mechanisms and Monte Carlo Dropout (MCD) for robust chili plant disease classification. VGG16 captures spatial and hierarchical features, while EfficientNetB0 ensures efficient, high‐accuracy learning. Attention enhances focus on disease‐relevant areas, and MCD improves robustness by estimating uncertainty. The study utilizes a chili plant disease dataset sourced from Kaggle, comprising 5000 images across five classes: Healthy, Leaf Curl, Leaf Spot, Whitefly, and Yellowish, after extensive data augmentation techniques, including rotation, flipping, zooming, and brightness adjustment, to improve model generalization. Feature extraction is performed using VGG16 and EfficientNetB0, followed by concatenation and refinement through attention mechanisms, enabling the model to focus on disease‐relevant features while suppressing background noise. MCD is integrated to estimate model uncertainty and mitigate overfitting. Experimental results demonstrate the superior performance of the proposed hybrid model. The concatenated VGG16 and EfficientNetB0 model achieved a classification accuracy of 99%, precision, and recall of 99%, surpassing individual model performances (VGG16: 96.8%, EfficientNetB0: 96.5%, and attention‐integrated variants reached up to 98%). The F1‐score reached 99% across all disease categories, ensuring high precision and recall. Compared to state‐of‐the‐art models like InceptionV3 (98.83%) and MobileNet (97.18%), the proposed hybrid model demonstrates improved classification accuracy and robustness. The study underscores the potential of deep learning‐based automated disease classification in precision agriculture, enabling early intervention and reducing reliance on chemical treatments. Future work aims to extend the approach to real‐time deployment on mobile and edge devices, integrate explainability techniques for enhanced interpretability, and explore federated learning for decentralized agricultural diagnostics.

## Graphical abstract

Introduced VGG‐EffAttnNet, a hybrid deep learning model combining VGG16 and EfficientNetB0 with an attention mechanism and Monte Carlo Dropout. Achieved 99% accuracy, precision, recall, and F1‐score across five chili plant disease classes. Outperformed individual models and state‐of‐the‐art methods, demonstrating superior robustness, generalization, and suitability for real‐time precision agriculture.

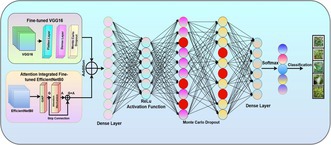

## 1 Introduction

Chili is a key crop that is grown worldwide, (Hussain and Abid 2011) accounting for a substantial portion of the agricultural economy and livelihood of millions of farmers. Chili is used extensively in culinary, pharmaceutical, and cosmetic applications owing to its high content of capsaicin, antioxidants, and vitamins. Large chili‐producing nations, (Mariyono and Sumarno 2015) such as India, China, Mexico, Thailand, and Indonesia, largely rely on its cultivation for local consumption and export. But chili farming is confronted with a number of challenges, (Islam et al. 2020) one of the most serious of which is plant disease, posing a threat to yield and quality. Different bacterial, fungal, and viral diseases, (Saxena et al. 2016) including anthracnose, powdery mildew, bacterial wilt, leaf curl virus, and Cercospora leaf spot, can severely affect chili production, causing economic losses of 20% to 80% in extreme cases. Early and precise diagnosis of such diseases is important for efficient disease management, (Shingote et al. 2022) increased yield, and eco‐friendly cultivation. Conventional chili plant disease diagnosis is mostly dependent on visual inspection by farmers and plant scientists (Ahmad Loti et al. 2021). Although this method has been followed for centuries, (Arsenovic et al. 2019) it has many drawbacks. Visual diagnosis of diseases is subjective and susceptible to human error, (González‐Pérez et al. 2011) particularly where symptoms are alike among diseases. The procedure is time‐consuming, demanding frequent field monitoring, which is not feasible for large farms. Moreover, agricultural experts are not easily accessible in most rural and distant farming areas, (Moyo 2024) slowing down the diagnosis process and enhancing the risk of misdiagnosis. Environmental conditions of light, humidity, (Sharma et al. 2025) and soil quality also make it difficult to identify, resulting in misclassifications of the disease. As a result of these difficulties, (Delai et al. 2024) farmers tend to use too many pesticides, which not only add to the cost of production but also degrade the environment, (Kanaparthi and Ilango 2023) making the soil infertile and polluting water sources. To overcome these challenges, (Seelam 2023) scientists have looked to machine‐based plant disease detection methods involving artificial intelligence and deep learning.

One of the biggest problems is the need for large, (Araujo et al. 2019) annotated datasets on which to train deep learning models successfully. Much research relies on small, (Pratap and Kumar 2023) constructed datasets that have a limited ability to generalize to alternative environmental conditions, (Anandamurugan 2025) resulting in overfitting and poor performance with unseen data. In addition, deep learning models are computationally complex and demand top‐end GPUs during training and inference, (Liu and Wang 2024) which makes it less feasible in mobile‐based and edge computing frameworks for smart agriculture. Overfitting is yet another issue, (Dhaka et al. 2021) especially when models are trained using constrained datasets with few regularization methods applied. Deep learning models are usually “black boxes,” not having the ability for interpretability, (Sultana et al. 2024) rendering it hard for farmers and agro experts to realize why they make specific predictions. Environmental variations, like light variation, noise in the environment, and foliage occlusions, (Ahmad et al. 2021) also affect model strength, degrading performance in practical scenarios. In an effort to tackle these shortfalls, this work presents a hybrid deep model combining various top‐of‐the‐line architectures for the classification of chili plant disease. The proposed approach marries the strength of EfficientNetB0 and VGG16 for both feature extraction and classification ability. EfficientNetB0 is added due to its computational speed and good feature extraction ability, which is apt for real‐time usage. VGG16, a popular CNN architecture, is added to utilize its powerful feature extraction ability. The combination model adopts a systematic flow that guarantees high accuracy and resilience.

First, input images are preprocessed through resizing, normalization, and augmentation to improve model generalization. The three deep learning models then extract the features, with EfficientNetB0 extracting light yet informative features, while VGG16 extracts spatial information. The three features are then combined by fusing the extracted features using a concatenation layer so that the model can take advantage of complementary strengths in every architecture. In addition to improving the classification performance, attention mechanisms are introduced to emphasize disease‐relevant features and suppress background noise with no relevance. Monte Carlo Dropout is also utilized to enhance uncertainty estimation and model robustness to provide confident predictions even with small datasets. Lastly, a fully connected layer followed by softmax classification is utilized to output the disease category. It is trained with categorical cross‐entropy loss and optimized with the Adam optimizer for better convergence. The key contributions of the study are as follows:

- Proposed a novel hybrid model (VGG‐EffAttnNet) that combines VGG16 and EfficientNetB0 to leverage both deep spatial and efficient semantic feature extraction for improved chili plant disease classification.
- Incorporated an attention module to enhance the model's ability to focus on disease‐relevant regions while suppressing irrelevant background noise, improving interpretability and classification precision.
- Applied Monte Carlo Dropout during inference to estimate prediction uncertainty and increase robustness, especially in cases with visually similar disease symptoms.
- Conducted thorough experiments including 5‐fold cross‐validation, inference time analysis, and comparison with state‐of‐the‐art models, demonstrating the model's superior accuracy (99%), stability, and feasibility for real‐time edge deployment.

The paper is structured as follows: Section 2 presents a literature review on recent advancements in deep learning‐based chili plant disease classification, highlighting related works and comparative studies. Section 3 describes the chili plant disease dataset used in this study, detailing dataset composition, augmentation techniques, and preprocessing steps. Section 4 outlines the selection of transfer learning models, discussing the rationale behind integrating VGG16 and EfficientNetB0 with attention mechanisms. Section 5 introduces the proposed hybrid model, detailing its architecture, feature extraction process, attention mechanisms, and Monte Carlo Dropout integration. Section 6 presents the results and performance evaluation, comparing individual model performances, hybrid feature fusion, and attention integration. Section 7 discusses an ablation study, analyzing the impact of architectural modifications on model accuracy and robustness. Section 8 provides a comparative analysis with state‐of‐the‐art methods, demonstrating the superiority of the proposed model. Finally, Section 9 concludes the study with a summary of key findings, potential real‐world applications, and directions for future research in deep learning‐based agricultural disease classification.

## 2 Literature Review

Literature review is represented in Table 1. Muthia and Salahuddin (2025) implemented the ResNet architecture on a self‐built dataset containing 2703 images and achieved an accuracy of 91%, with precision and recall both at 0.94, and an F1‐score of 0.93. This performance is decent for a baseline model. However, the limitation of this study lies in its relatively lower accuracy when compared with more advanced models. Additionally, ResNet, being a deep model, may suffer from high computational complexity, making it less ideal for deployment in resource‐constrained environments such as small farms. Gulzar and Ünal (2025) proposed a PL‐DenseNet model on the large‐scale DiaMOS Plant Dataset comprising 7337 images. The model achieved exceptional results: 99.18% accuracy, 98.83% precision, 99.06% recall, and an F1‐score of 0.9358. Despite these high metrics, the limitation of this work is its dependence on a large and high‐quality dataset. In real‐world agricultural settings, obtaining such large annotated datasets is often challenging, making the model less transferable to practical applications without fine‐tuning. Gulzar and Ünal (2025) also explored DenseNet121 on a smaller dataset of 1214 images, achieving a promising accuracy of 99.45%. However, the limitation is the lack of other evaluation metrics such as precision, recall, and F1‐score. Without these, it is difficult to assess whether the model is equally effective across all disease classes, especially in cases of data imbalance. Gulzar and Ünal (2025) further used the MIV‐PlantNet deep learning technique on the Saudi Arabia FloraD dataset, achieving 99% accuracy. While the result is impressive, the limitation is the absence of key metrics like precision and recall, which are crucial for evaluating performance in multiclass settings. Additionally, no information was provided about the dataset's diversity, which questions the model's generalizability. Hamim and Jony (2024) utilized the MobileNet model on a small dataset of 300 images and reported 97.18% accuracy. The limitation of this approach is the very small dataset size, which increases the risk of overfitting. The model may perform well on test data from the same distribution but may fail when introduced to real‐world variability in leaf appearance or background noise. Srinivasulu and Maiti (2024) introduced the RNDDNet model and applied it to 3800 chili images, achieving 98.09% accuracy, 97% precision, and an F1‐score of 97.25%. The main limitation of this study is the complexity of the RNDDNet architecture, which may pose challenges in terms of model interpretability and computational requirements, especially for on‐field deployment using edge devices. Naresh et al. (2024) adopted the SEDCNN architecture on a self‐built chili dataset with 2265 images, reaching 97% accuracy. Although the result is commendable, the study did not report precision, recall, or F1‐score, which are essential for understanding model performance in class‐specific disease identification. Murinto and Pujiyanta (2024) employed VGG16 on a dataset of only 250 images, achieving 94% accuracy. The limitation here is again the very small dataset, which limits the model's robustness. Moreover, VGG16 is known for its large number of parameters, which can make training computationally intensive and less suited for low‐power devices. Li et al. (2024) applied the MCSAM model to 500 images and achieved 91.2% accuracy. The study suffers from both low dataset size and lack of other performance metrics. Without a proper evaluation of precision and recall, the reliability of the model in multiclass settings remains questionable. Hanafi et al. (2024) used ResNet50 on a large dataset of 9022 images and achieved 95% accuracy. While the model performed well, the limitation lies in the absence of precision, recall, and F1‐score. These are critical for understanding how well the model identifies specific disease categories. Aminuddin et al. (2024) trained EfficientNetB0 on the Plant Village dataset with 3000 images, achieving 97.5% accuracy, 92% precision, and 0.92 specificity. However, the recall was only 0.97, and the model showed some misclassifications. The limitation lies in its moderate performance for certain classes, suggesting potential bias in class distribution or feature extraction inefficiencies. Gulzar et al. (2024) implemented InceptionV3 on a dataset of 5513 images and achieved 98.73% accuracy. Though highly accurate, the study lacks class‐wise metrics such as recall or F1‐score, which limits its applicability for disease classification tasks where false negatives can have significant impacts. Alkanan and Gulzar (2024) applied MobileNetV2 to a massive dataset of 21,662 images and achieved 96% accuracy. The key limitation is that lightweight models like MobileNetV2 may sacrifice some classification granularity, particularly in detecting subtle visual differences between similar diseases. Seelwal et al. (2024) proposed a hybrid deep learning model using the BRRI and IRRI datasets. However, the major limitation is the absence of performance metrics such as accuracy, precision, or recall. Without these, it's not possible to assess or compare the model's effectiveness. Vasavi et al. (2023) used YOLOv5 on a self‐built dataset with 210 images and achieved 75.64% accuracy, which is significantly lower than other studies. The major limitation is the small dataset size and the lower model performance, making it unsuitable for practical applications without further optimization and data expansion. Rahadiyan et al. (2023) utilized EfficientNetB4 on 2000 images and reported 92% accuracy. While the result is promising, the limitation is again the absence of detailed class‐wise performance metrics, which are vital for evaluating disease detection in real‐world agricultural scenarios. Rahadiyan et al. (2023) experimented with CNN, SVM, and MLP on 5166 images. CNN achieved 97.76% accuracy, while SVM and MLP showed lower performance. A limitation here is that while CNN performed well, traditional methods like SVM and MLP lagged significantly, showing that classical models may not be suitable for image‐based disease classification. Ramadhani et al. (2023) applied MobileNetV2 to 2494 images, reaching 90% accuracy and an F1‐score of 92%. Though MobileNetV2 is efficient, its accuracy is relatively low compared to other deep models, indicating potential trade‐offs between model size and performance. Shao et al. (2024) used the SPA‐BP technique and achieved 92.3% accuracy, but the limitation lies in not providing dataset details or performance across different disease categories. This makes it difficult to evaluate the model's overall effectiveness and reliability. Chaitanya et al. (2023) implemented ResnetCNN and reported 86.1% accuracy, which is considerably lower than other models. The limitation is likely due to limited model optimization or inadequate dataset quality, reducing its suitability for field applications. Gulzar et al. (2023) used a Pretrained Xception model on 3018 RGB images and astonishingly reported 100% accuracy. The primary limitation here is potential overfitting or dataset leakage. A perfect score is rare and often indicates that the model has seen test data or lacks real‐world variability. Naik et al. (2022) applied SECNN to 5100 chili leaf images, achieving 98.82% accuracy. While this is a strong result, the study did not evaluate the model's resilience to variations such as different lighting conditions or background clutter. Patil and Lad (2022) compared SVM and KNN on a dataset of 2500 images, achieving 87.04% and 94.04% accuracy, respectively. These classical models performed worse than deep learning models, highlighting their limitation in handling complex, high‐dimensional image data. Rozlan and Hanafi (2022) utilized InceptionV3 on 3000 images, achieving 98.83% accuracy, with 99% precision, 99% recall, and 0.99 F1‐score. Although results are impressive, the limitation is that the model was not validated on external datasets, which is crucial for assessing generalizability in real‐world applications.

**TABLE 1:** Literature review.

References	Dataset	Techniques	No. of images	Results
Muthia and Salahuddin (2025)	Self‐built dataset	ResNet	2703	Accuracy: −91% Precision: −0.94% Recall: −0.94% F1 Score: −0.93%
Gulzar and Ünal (2025)	DiaMOS Plant dataset	PL‐DenseNet	7337	Accuracy: −99.18% Precision: −98.83% Recall: −99.06% F1 Score: −9358%
Gulzar et al. (2024)		DenseNet121	1214	Accuracy: −0.9945%
Amri et al. (2024)	SaudiArabiaFlora D	MIV‐PlantNet deep learning		Accuracy: −99%
Hamim and Jony (2024)	Chili leaf Disease	MobileNet	300	Accuracy: −97.18%
Srinivasulu and Maiti (2024)	Chili plant disease	RNDDNet	3800	Accuracy: −98.09% Precision: −97% Recall: −97.25% F1 Score: −97.25%
Naresh et al. (2024)	Self‐Built Chili Dataset	SEDCNN	2265	Accuracy: −97%
Murinto and Pujiyanta (2024)	Chili plant disease	VGG16	250	Accuracy: −94%
Li et al. (2024)	Chili plant Disease	MCSAM	500	Accuracy: −91.2%
Hanafi et al. (2024)	Self	Resnet50	9022	Accuracy: −95%
Aminuddin et al. (2024)	Plant–village–website	EfficientNet‐b0	3000	Accuracy: −97.5% Recall: −0.97% Specification: −0.92% Precision: −92% F1‐Score: −0.94%
Gulzar (2024)	—	InceptionV3	5513	Accuracy: −98.73%
Alkanan and Gulzar (2024)	—	MobileNetV2	21,662	Accuracy: −96%
Seelwal et al. (2024)	BRRI, IRRI	hybrid deep learning		
Vasavi et al. (2023)	Self	YOLOv5	210	Accuracy: −75.64%
Pratap and Kumar (2023)	Self	EfficientNetB4	2000	Accuracy: −92%
Rahadiyan et al. (2023)	Self	CNN	5166	Accuracy: −97.76% Accuracy: −90.55% Accuracy: −89.70%
		SVM		
		MLP		
Ramadhani et al. (2023)	Self	MobileNetv2	2494	Accuracy: −90% F1‐Score: −92%
Shao et al. (2024)	Self	SPA‐BP	—	Accuracy: −92.3%
Gulzar et al. (2023)	Self	ResnetCNN	—	Accuracy: −86.1%
Chaitanya et al. (2023)	Self	Pretrained Xception	3018RGB	Accuracy: −100%
Naik et al. (2022)	Self‐built chili leaf dataset	SECNN	5100	Accuracy: −98.82%
Patil and Lad (2022)	Self	SVM	2500	Accuracy: −87.04% Accuracy: −94.04%
		KNN		
Rozlan and Hanafi (2022)	Self	InceptionV3	3000	Accuracy: −98.83% Precision: −99.00% Recall: −99.00% F1‐Score: −0.99%

Recent developments in deep learning have significantly advanced plant disease classification. Hu et al. (2019) introduced an improved CNN for tea leaf disease detection, while Heng et al. (2024) enhanced this approach using hybrid pooling strategies. Li et al. (2025) proposed AO‐DETR, an attention‐based model designed for object detection, which can be adapted for precise localization in plant disease tasks. Zhang et al. (2024) developed a multiview learning method for molecular prediction, offering potential for complex plant image analysis. Bao et al. (2022) applied AX‐RetinaNet to identify tea leaf diseases more effectively using attention mechanisms. Liao et al. (2024) explored meta‐learning to adapt models across domains, which is valuable in varying agricultural environments. Wang et al. (2025) introduced a multimodal knowledge transfer model (MDKAT) that could help handle diverse plant image features. Zeng et al. (2022) proposed LDSNet for lightweight corn disease detection, while He et al. (2025) examined multispectral reflectance of plant canopies for better feature understanding. Rashid et al. (2024) integrated IoT and deep learning for early corn disease detection using multiple models. Al‐Selwi et al. (2025) addressed RGB image complexity in cluttered scenes, with relevance to noisy field conditions in agriculture. Subburaj et al. (2025) presented Plantention, an attention‐based lightweight model effective across various crop diseases. Finally, Yadav et al. (2021) demonstrated CNN‐based identification of bacteriosis in peach leaves. These works collectively highlight the integration of deep learning, attention mechanisms, and domain adaptation in smart agricultural diagnostics. The summary of the above‐reviewed literature is presented in Table 1: Literature review.

## 3 Methods and Materials

The study utilizes the Chili Plant Disease Dataset sourced from Kaggle, categorized into five classes: Healthy, Leaf Curl, Leaf Spot, Whitefly, and Yellowish, and is divided into training, validation, and test sets. To improve model generalization and performance, various data augmentation techniques such as rotation, flipping, zooming, brightness adjustment, and contrast enhancement are applied. A hybrid deep learning approach integrates VGG16 and EfficientNetB0 for feature extraction and classification; VGG16 extracts hierarchical features through sequential convolutional layers, while EfficientNetB0 employs MBConv blocks with Squeeze‐and‐Excitation (SE) attention for efficiency. Their features are concatenated, followed by fully connected layers and Monte Carlo Dropout (MCD) for uncertainty estimation, using softmax for multiclass classification. Model performance is assessed using accuracy, precision, recall, F1‐score, loss, and AUC, where accuracy indicates overall correctness, precision and recall evaluate disease identification effectiveness, the F1‐score balances precision and recall, loss tracking monitors convergence, and AUC measures classification performance across different thresholds.

### 3.1 Input Dataset

**FIGURE 1:**
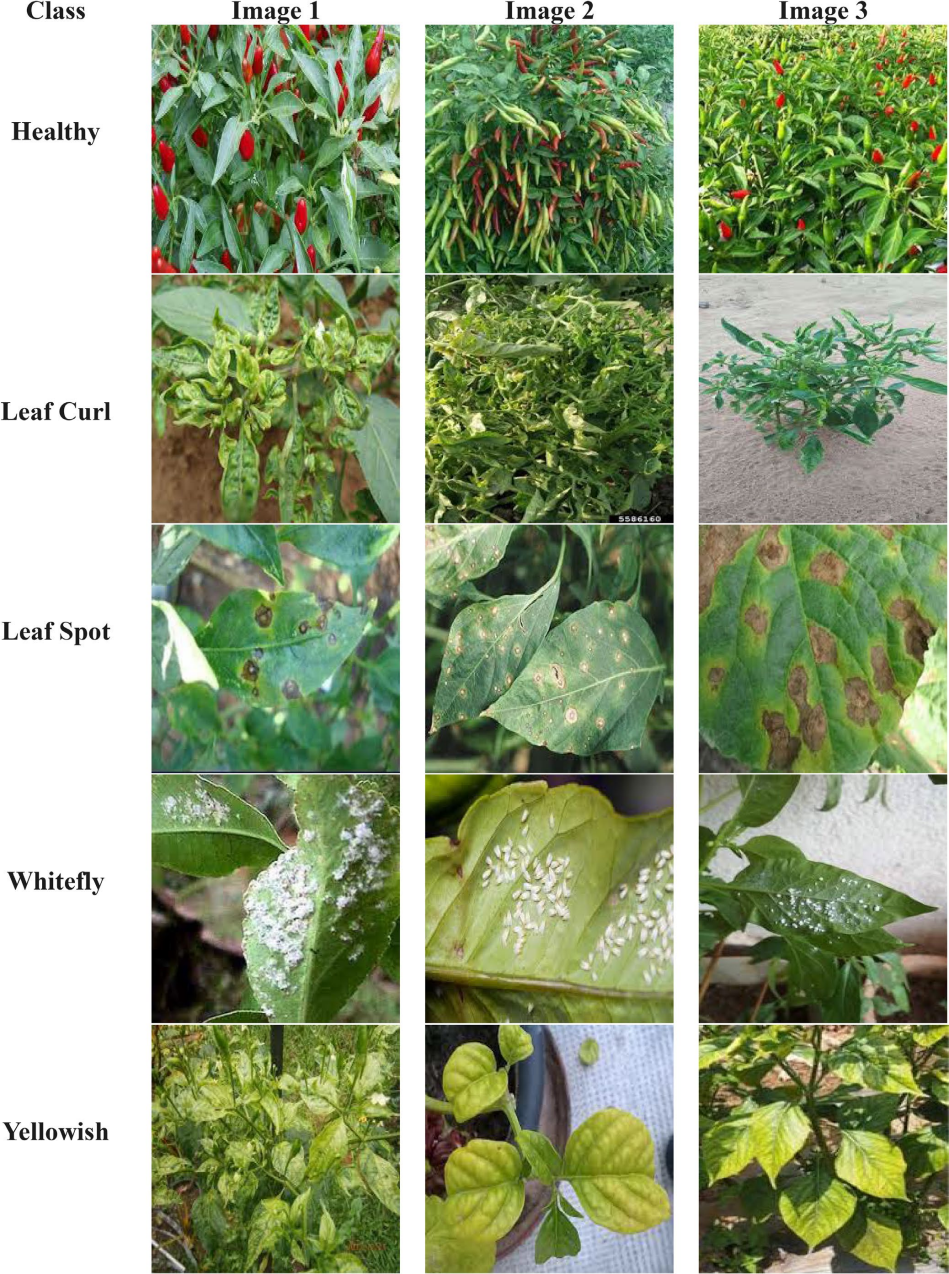
Input dataset.

The input dataset for chili plant disease classification is sourced from Kaggle (Ramadhani et al. 2023) as shown in Figure 1, an open‐access online platform providing high‐quality datasets for machine learning research. Each of the five classes: Healthy, Leaf Curl, Leaf Spot, Whitefly, and Yellowish, has 100 photographs in this 500‐image dataset. Training (80 images per class), Validation (10 images per class), and Testing (10 images per class) comprise the dataset to guarantee an efficient mix for model training, fine‐tuning, and evaluation. Data augmentation methods include rotation, flips, zooming, brightness modification, and contrast enhancement, which are used to improve model generalization and stop overfitting. These augmentations bring variances in the dataset, therefore strengthening the model's resilience to visual distortions and environmental changes. This dataset helps to construct extremely accurate chili plant disease classification systems by using deep learning‐based techniques including hybrid models and CNNs. Such developments can greatly help early disease detection, precision agriculture, and efficient crop health monitoring, thereby enhancing yield management and sustainable farming methods.

### 3.2 Data Augmentation Techniques

**FIGURE 2:**
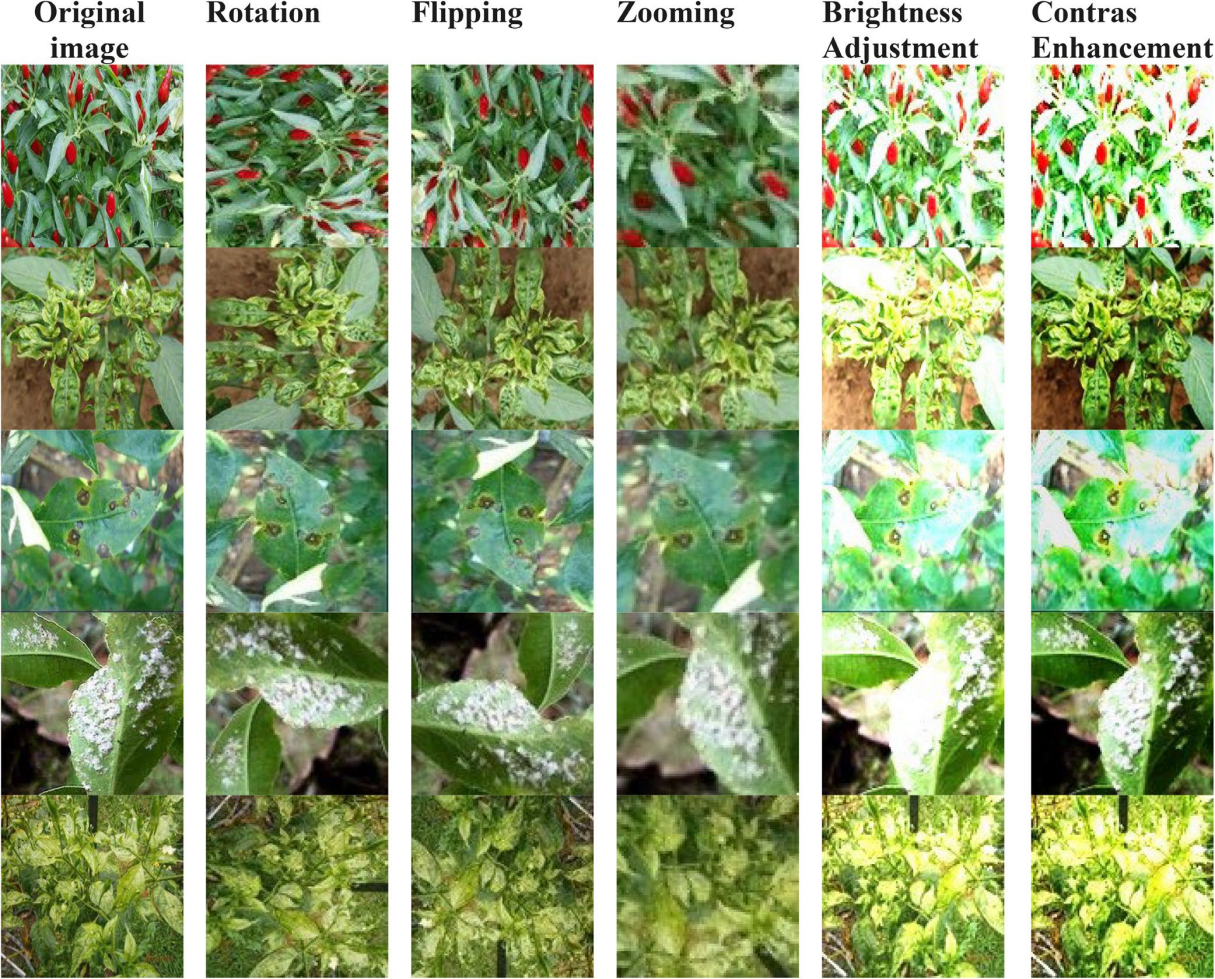
Sample images of data augmentation.

Figure 2 depicts some of the data augmentation techniques applied to images of chili plant diseases to enhance dataset diversity and robustness for training deep learning models. All images were resized to a uniform resolution of 224 × 224 pixels to standardize input dimensions. All the augmentation methods: rotation, flipping, zooming, brightness adjustment, and contrast are used to enhance model generalization and accuracy. Rotation alters the orientation of the leaf by angles in a way that the model can recognize disease patterns from numerous angles. Horizontal and vertical flipping both change the orientation of the image, thus ensuring that the model is not biased towards any single leaf position. Through magnification or reduction of specific leaf sections, zooming alters the size of the images thus enabling the model to focus on regions affected by disease. Through the imitation of different lighting conditions, brightness change enables the model to be resilient to variations in natural light. Finally, by modifying the intensity fluctuations between affected and unaffected leaf sections, contrast enhancement enhances symptom visibility. These enhancement techniques artificially expand the data set, thereby enhancing generalization and allowing deep learning algorithms to detect chili plant diseases with greater precision. Once augmentation has been applied, the size of the dataset grows dramatically. The 10 augmentations for each image yield a dataset of 5000 images. In Table 2, data augmentations are represented. Divided among five classes: Healthy, Leaf Curl, Leaf Spot, Whitefly, and Yellowish, the distribution at the end includes 500 test photos, 4000 training images, and 500 validation images. Tenfold augmentation ensures 100 test images per class, 800 training images per class, and 100 validation images per class. Sourced from Kaggle, such a large dataset enhances the accuracy of the model in classifying chili plant diseases under varying conditions, thus enhancing real‐world applicability.

**TABLE 2:** Augmented dataset.

Class	Before augmented	After augmented	Before augmented	After augmented	Before augmented	After augmented
	Training		Testing		Validation	
Healthy	80	800	10	100	10	100
Leaf Curl	80	800	10	100	10	100
Leaf Spot	80	800	10	100	10	100
Whitefly	80	800	10	100	10	100
Yellowish	80	800	10	100	10	100
Total	400	4000	50	500	50	500

## 4 Selection of Transfer Learning Model

Developing an accurate and efficient deep learning pipeline depends critically on the choice of transfer learning models. As Table 3 shows, VGG16 and EfficientNetB0 have been selected for their demonstrated efficacy, architectural advantages, and fit with the goal of chili plant disease classification. Using a compound scaling approach that proportionately changes network depth, width, and resolution, EfficientNetB0 guarantees a balance between high accuracy and low processing time. Strong feature extraction from challenging datasets made possible by its lightweight MBConv blocks, supplemented with Squeeze‐and‐Excitation (SE) attention, qualifies it for resource‐limited settings. Conversely, VGG16 offers strong and consistent performance for image classification activities since it is well known for its hierarchical feature extraction using successive convolutional layers. The integration of both models guarantees effective deep feature extraction and computational performance. Derived from massive datasets such as ImageNet, the pretrained weights of these models enable efficient transfer learning by capturing generalized properties fit for particular applications. This strategic model choice balances speed and accuracy to maximize performance, making it ideal for practical uses in the diagnosis of agricultural diseases.

**TABLE 3:** Specification parameters of VGG16 and EfficientNetB0.

Specification	VGG16	EfficientNetB0
Architecture	Sequential Convolutional Layers	Compound Scaling (depth, width, resolution)
Number of layers	16 layers	~82 layers
Model size	528 MB	5.3 MB
Number of parameters	138 million	5.3 million
Input image size	224 × 224	224 × 224
Top‐1 accuracy	71.5% (ImageNet)	77.1% (ImageNet)
FLOPs	15.5 billion	390 million
Training Speed	Slower due to more parameters	Faster due to fewer parameters
Strengths	Strong feature extraction, widely used	High efficiency, lightweight, balanced accuracy
Pretrained weights	Available (ImageNet, etc.)	Available (ImageNet, etc.)

Compared to other deep learning models, VGG16 and EfficientNetB0 offer an optimal balance of accuracy and computational efficiency. EfficientNetB0 offers great accuracy with few parameters, hence it is perfect for real‐world deployment even if VGG models demand more parameters and are computationally expensive. While MobileNet is lightweight but lacks the accuracy of EfficientNetB0, other models as DenseNet attain great accuracy but are computationally demanding. Combining the great feature extraction of VGG16 with the efficiency of EfficientNetB0 guarantees consistent and scalable disease classification in chili plants.

## 5 Proposed VGG‐EffAttnNet Model

**FIGURE 3:**
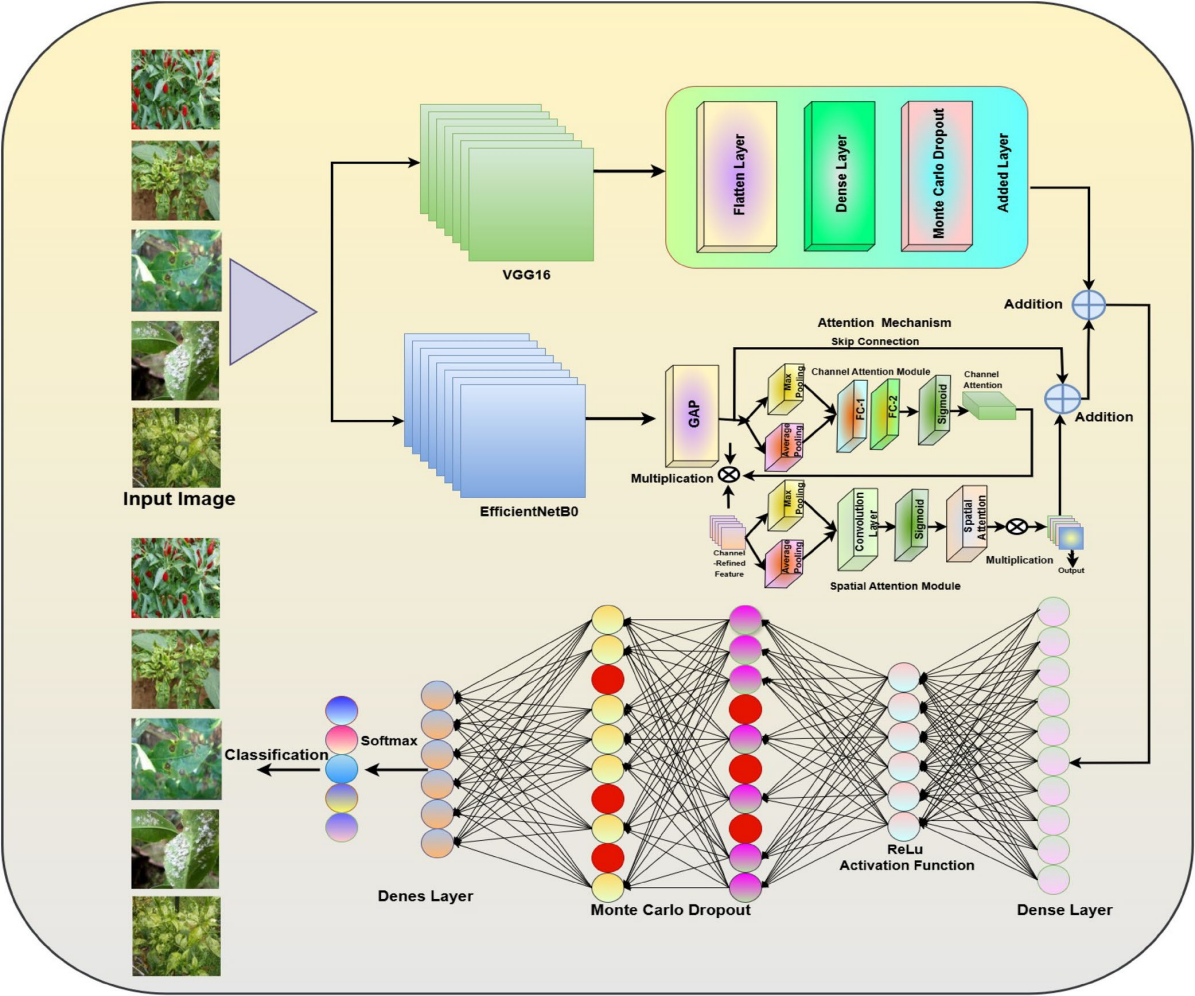
Architecture of VGG‐EffAttnNet model.

Figure 3 represents the architecture of a hybrid deep learning model for chili plant disease classification incorporating VGG16 and EfficientNetB0 with attention mechanisms and Monte Carlo Dropout. The images as input are passed through two independent deep learning models: VGG16, which specializes in hierarchical feature extraction, and EfficientNetB0, which utilizes efficient scaling and global feature representation. The VGG16 pathway consists of convolutional layers and flattening, dense layers, and MCD to estimate uncertainty for better predictions. The EfficientNetB0 pathway adds Global Average Pooling and an attention mechanism to fine‐tune the extracted features before combining them through a skip connection. Both models' outputs are concatenated and passed through fully connected layers with ReLU activation. To improve generalization and avoid overfitting, Monte Carlo Dropout is introduced prior to the last Softmax classification layer, which classifies the input into various chili plant disease categories. Monte Carlo Dropout is applied during inference to perform multiple stochastic forward passes, enabling the model to estimate prediction uncertainty. The improves robustness by preventing overconfident predictions and enhancing reliability, especially in ambiguous or overlapping disease cases. The combination of VGG16's ability to learn textures and EfficientNetB0's attention‐based feature extraction enhances the accuracy of the model, thus making it an effective tool for plant disease diagnosis.

### 5.1 Fine‐Tuned VGG16 Model

**FIGURE 4:**
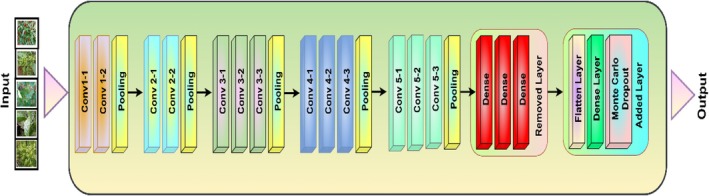
Architecture of fine‐tuned VGG16 model.

Figure 4 represents the fine‐tuned VGG16 architecture used for chili plant disease classification, incorporating Monte Carlo Dropout for uncertainty estimation. The model processes input images through sequential convolutional layers (Conv1 to Conv5), where each block applies convolutional operations followed by pooling layers to extract hierarchical features. After the final pooling layer, the fully connected layers are shown, where some layers are removed or modified to adapt to the classification task. The modified architecture includes a Flatten layer, a dense layer, and Monte Carlo Dropout, enhancing feature learning and reducing overfitting. The fine‐tuning ensures the VGG16 model efficiently classifies various chili plant diseases with improved accuracy and generalization.

Mathematical Equations Representing the Convolutional Neural Network with Monte Carlo Dropout and Attention Mechanism: The provided image represents a deep convolutional neural network with multiple convolutional layers, pooling operations, fully connected layers, Monte Carlo Dropout, and an attention mechanism. Below are the step‐by‐step mathematical equations corresponding to this architecture.

1. Input representation given in Equation (1): Let $X\in R^{H\times W\times C}$ represent the input image, where H = height of the input, W = width of the input, C = number of channels (e.g., 3 for RGB images)

(1) $$X=\left\{X_{1,}X_{2,\ldots \ldots ,}X_{c}\right\}$$

2 Convolutional layers: The image passes through multiple convolutional layers. A convolution operation can be defined as shown in Equation (2):

(2) $$X_{l}^{,}=\sigma \;\left(W_{l}*X_{l-1}+b_{l}\right)$$

where $X_{l-1}$ = Input to the l‐th convolutional layer, $W_{l}$ = Convolutional kernel (filter), *= Convolution operation, $b_{l}$ = Bias term, σ = Activation function (e.g., ReLU), Each set of convolutional layers (Conv 1–1, Conv 1–2, Conv 2–1, etc.) applies this transformation.

3 Pooling layers: After each set of convolutional layers, a pooling layer is applied to reduce the spatial dimensions.

- Max pooling represents in Equation (3):

(3) $$P_{l}=\max_{i,j\in k}X_{l}^{\prime }\left(i,j\right)$$

where K is the pooling kernel size. Pooling layers downsample the feature maps to reduce computational complexity.

4 Flatten layer: As shown in Equation 4

(4) $$F=\text{Flatten}\;\left(P^{\left(5\right)}\right)$$

where $P^{\left(5\right)}$ =Pooled output from the last convolutional block (Conv5), F = one‐dimensional feature vector.

5 Fully connected layers (Dense layers): After passing through all convolutional and pooling layers, the feature maps are flattened and passed through fully connected (dense) layers. The process is defined in Equation (5):

(5) $$D=\sigma \;\left(W_{d}\cdot F+b_{d}\right)$$

where $W_{d}$: Weight matrix for the dense layer, $b_{d}$: Bias vector, σ: Activation function (ReLu), D: Output of the dense layer.

6 Monte Carlo Dropout: Monte Carlo Dropout is applied to simulate Bayesian inference as shown in Equation 6

(6) $$D_{\mathrm{MCD}}=\text{Dropout}\;\left(D,\text{rate}=p,\text{training}=\text{true}\right)$$

where D, rate: is the dropout rate. training=true: Ensures dropout during inference, $D_{\mathrm{MCD}}$: Output after Monte Carlo Dropout.

7 Output Layer‐Softmax Classification: as shown in Equation 7

(7) $$\hat{Y}=\text{Softmax}\;\left(W_{{}^{\circ}}.D_{\mathrm{MCD}}+b_{{}^{\circ}}\right)$$

where $W_{{}^{\circ}}$: Weights of the output layer, $b_{{}^{\circ}}$: Bias term of the output layer, $\hat{Y}$: Predicted class probability distribution over N Classes.

8 Output layer (Softmax Classification): The final classification layer uses a softmax activation as given in Equation 8

(8) $$\hat{Y}=\text{Softmax}\;\left(W_{{}^{\circ}}.\text{Dropout}\;\left(D\right)+b_{{}^{\circ}}\right)$$

where $W_{{}^{\circ}\prime }b_{{}^{\circ}}$ = output layer weights and bias, $\hat{Y}$ = Probability distribution over N classes.

Final model representation in Equation (9):

(9) $$\hat{Y}=\text{Softmax}\;\left(W_{{}^{\circ}}\left(D_{\mathrm{MCD}},D_{\mathrm{MCD}}\right)+b_{{}^{\circ}}\right)$$

### 5.2 Attention‐Integrated Fine‐Tuned EfficientNetB0

**FIGURE 5:**
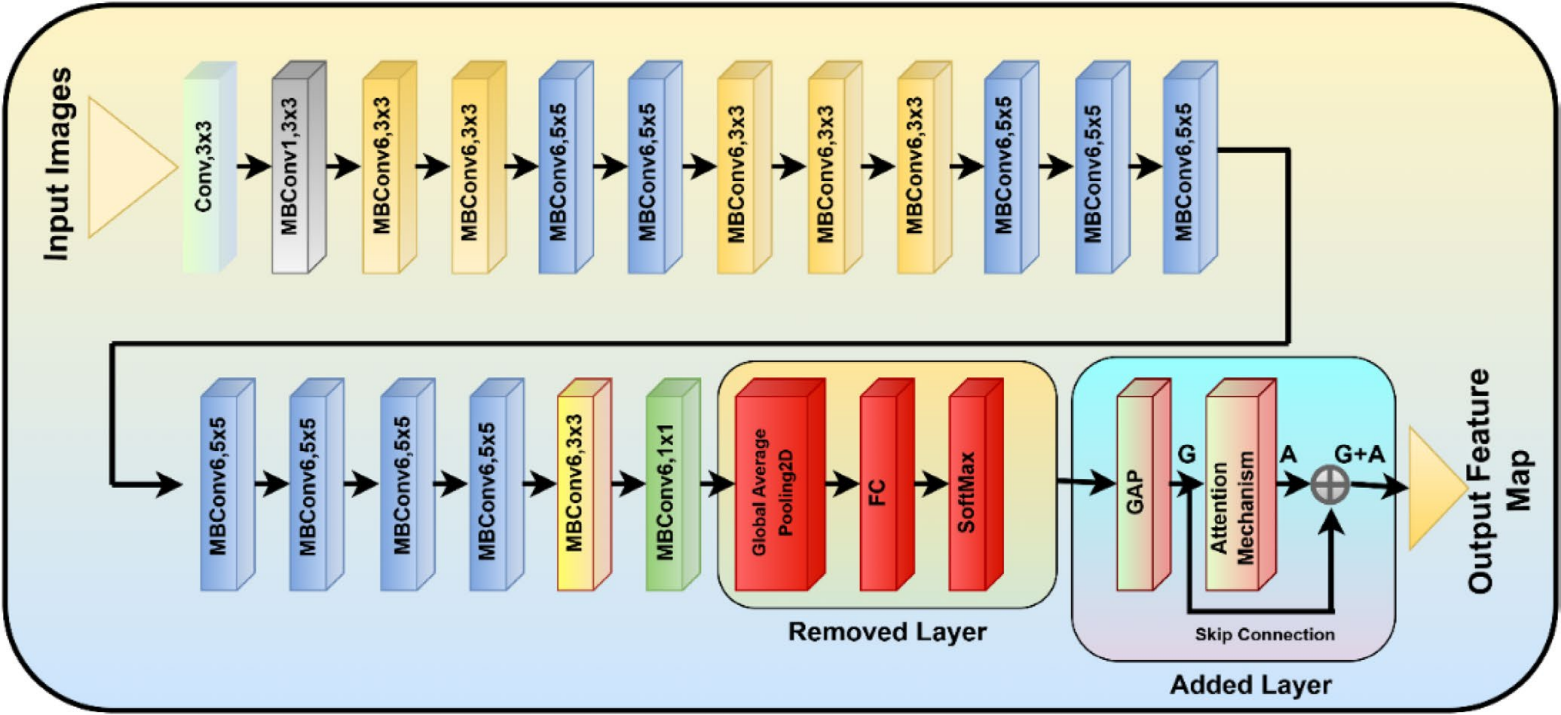
Architecture of attention‐integrated fine‐tuned EfficientNetB0 Model.

Figure 5 illustrates the architecture of the Attention‐Integrated fine‐tuned EfficientNetB0 model developed for automated chili plant disease classification. The architecture starts with an input image that passes through a series of convolutional layers and Mobile Inverted Bottleneck Convolution (MBConv) blocks, which are the core components of EfficientNetB0, designed for efficient feature extraction using depthwise separable convolutions and squeeze‐and‐excitation modules. The original classification layers of EfficientNetB0, including the Global Average Pooling (GAP), Fully Connected (FC), and Softmax layers – are removed to allow model customization. The output feature maps from the final convolutional block are passed through a Global Average Pooling 2D layer to create a compact feature vector. This vector (denoted as G) is fed into an Attention Mechanism that learns to emphasize important features (denoted as A). A skip connection is applied to merge the original and attention‐enhanced features using an Add layer (G + A). The resulting output feature map is then used for concatenation in the hybrid model. This fine‐tuning strategy enhances both the efficiency and accuracy of disease classification by leveraging the power of attention without altering the EfficientNetB0 backbone structure.

1. Input representation: Let $X\in R^{H\times W\times C}$ represent the input image, where H = height of the input, W = width of the input, C = 3: Number of channels (RGB) is presented in Equation (10):

(10) $$X=\left\{X_{1,}X_{2,\ldots \ldots ,}X_{c}\right\}$$

2 Feature extraction by EfficientNetB0 backbone: as shown in Equation 11

(11) $$F=F_{\text{EffNet}}\left(X\right)$$

where $F_{\text{EffNet}}$: EfficientNetB0 convolutional blocks (MBConv), $F\in R^{h\times w\times d}$: Output feature map.

3 Global average pooling (GAP) and ## (FC) Layers

- GAP reduces feature maps to a single value per channel, as present in Equation (12):

(12) $$G=\mathrm{GAP}\left(F\right)=\frac{1}{h\cdot w}\sum_{i=1}^{h}\sum_{j=1}^{w}F\left(i,j\right)$$

where $G\in R^{d}$:1D global feature vector.

4 Attention mechanism: as shown in Equation 13

(13) $$A=\text{Attention}\;\left(G,G\right)$$

where Both Query and value are the same (G), as used in the code, computes an attention‐weighted feature vector, internally represent Equation (14):

(14) $$\propto =\text{softmax}\;\left(\tanh\left(W_{q}G+W_{k}G\right)\right)$$

where G is used as both the Query and Key, $W_{q},W_{k}$: Learnable weight matrices for the query and key, tanh: Nonlinear activation, ∝: Attention weight‐tells the model where to focus, softmax: Concerts scores into probabilities (weight).

Apply Attention Weights to the value: as shown in Equation (15):

(15) A=∝.G

where ∝: Learning attention weights, G: Value vector (same as query in your case), A: Output of the attention layer—the refined feature vector.

5 Skip connection & addition layer: A skip connection applied between earlier and later MBConv blocks is present in Equation 16

(16) Y=G+A

where $Y\in R^{d}$: Combined original and attention features.

6 Output layer (Feature Map). Finally, the processed feature maps are concatenated or added before passing to the output, which is present# in Equation 17

(17) $${\text{Output}}_{\text{EffNet}}=Y=G+A$$

### 5.3 Attention Mechanism

**FIGURE 6:**
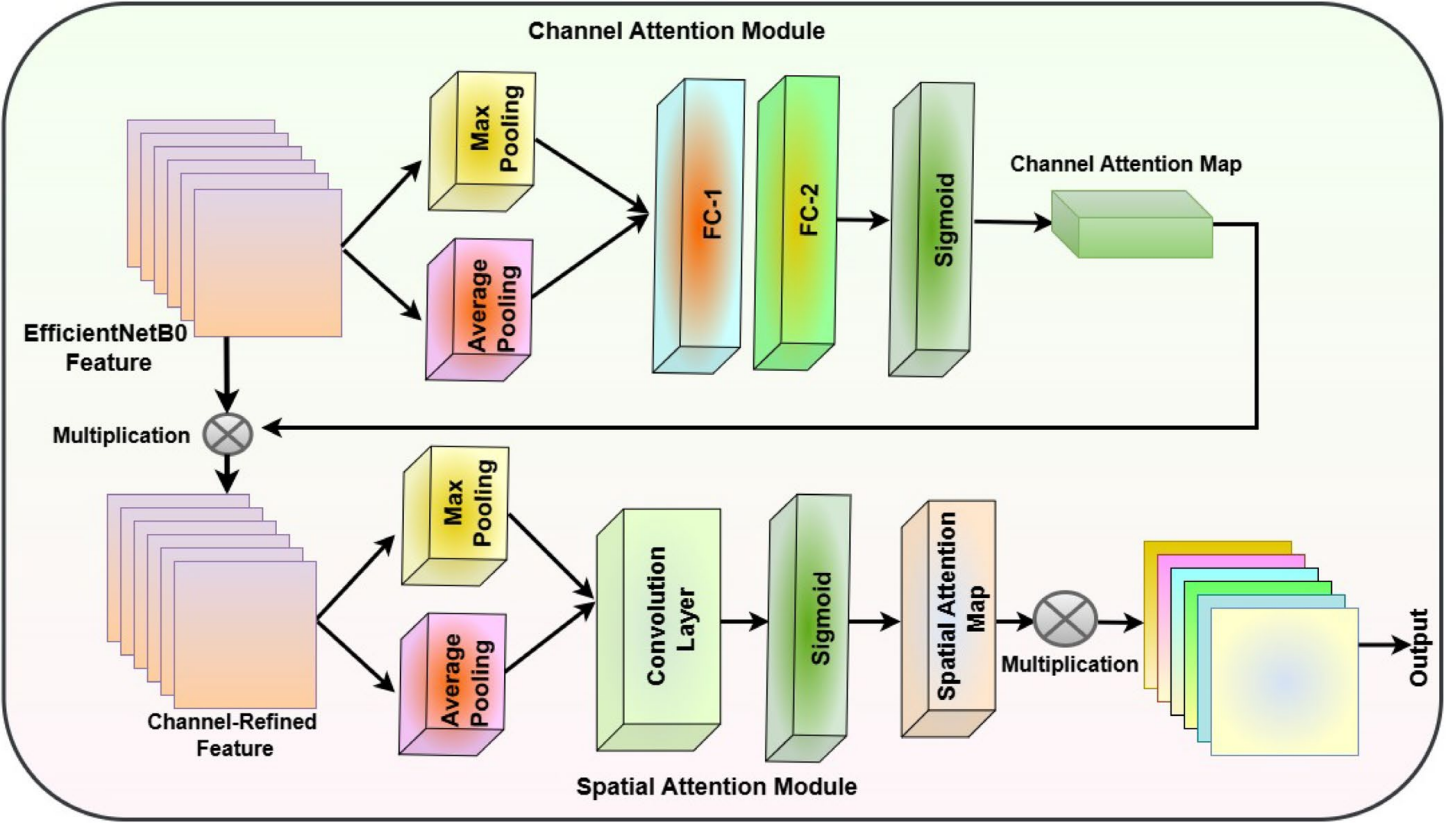
Architecture of attention mechanism.

Figure 6 illustrates an Attention Mechanism that enhances convolutional feature maps through two sequential modules: the Channel Attention Module and the Spatial Attention Module. In the Channel Attention Module, the input feature map undergoes both Max Pooling and Average Pooling to capture different contextual information. The pooled outputs are passed through two fully connected layers, labeled FC‐1 and FC‐2, followed by a Sigmoid activation, which generates the Channel Attention map. This map is then multiplied with the original feature map to refine it along the channel dimension. The resulting Channel‐Refined Feature is forwarded to the Spatial Attention Module. Here, the refined feature map is processed again through Max Pooling and Average Pooling. These outputs are passed through a Convolution Layer and Sigmoid activation to produce the Spatial Attention map, which is multiplied with the input to emphasize important spatial regions. The final output feature map thus benefits from both channel‐wise and spatial‐wise attention refinements, enhancing the representational power of the features extracted from the original input (Figure 6).

1. Channel attention module

- Spatial pooling operations: as shown in Equation (18).

(18) $$F_{\mathrm{Avg}}^{c}=\text{AvgPool}\left(F\right),F_{\max}^{c}=\text{MaxPool}\left(F\right)$$

where $F_{\mathrm{Avg}}^{c},F_{\max}^{c}\in R^{1\times 1\times c}$

- Fully connected layers + Sigmoid (Separate Paths) as shown in Equation (19):

(19) $$M_{c}^{\mathrm{Avg}}=\sigma \left(FC_{2}\left(FC_{1}\left(F_{\mathrm{Avg}}^{c}\right)\right)\right)$$

(20) $$M_{c}^{\mathrm{Max}}=\sigma \left(FC_{2}\left(FC_{1}\left(F_{\mathrm{Max}}^{c}\right)\right)\right)$$

- Generate channel attention map

The figure does not show combination (like summation), so we represent the channel attention as either of the two or both used independently: as represented in Equation (21).

(21) $$M_{c}=\text{Channel Attention}\;\mathrm{Map}\;\left(\text{from either path}\right)$$

- Apply channel attention: as shown in Equation (22).

(22) $$F^{\prime }=M_{c}⊙F$$

where ⊙ Denoted element‐wise multiplication, $F^{\prime }$: Channel‐refined feature map.

2 Spatial attention module

- Channel pooling operations: as represented in Equation (23).

(23) $$F_{\mathrm{avg}}^{s}={\text{AvgPool}}_{\text{channel}}\left(F^{\prime }\right),F_{\max}^{s}={\text{MaxPool}}_{\text{channel}}\left(F^{\prime }\right)$$

where $F_{\mathrm{avg}}^{s}$, $F_{\max}^{s}\in R^{H\times W\times 1}$

- Concatenation + convolution + sigmoid: as shown in Equation (24).

(24) $$M_{s}=\sigma \left(\text{Conv}\;\left(\left[F_{\mathrm{avg}}^{s};F_{\max}^{s}\right]\right)\right)$$

where [.;.]: concatenation along the channel axis, Conv:2D convolution layer, $M_{s}\in R^{H\times W\times 1}$: spatial map.

- Apply spatial attention: as shown in Equation (25).

(25) $$F^{"}=M_{s}⊙F^{\prime }$$

3 Final output represented in Equation 26

(26) $$\text{Output}=F^{"}\in R^{H\times W\times 1}$$

### 5.4 Concatenation of Fine‐Tuned VGG16 and Attention Integrated Fine‐Tuned EfficientNetB0

**FIGURE 7:**
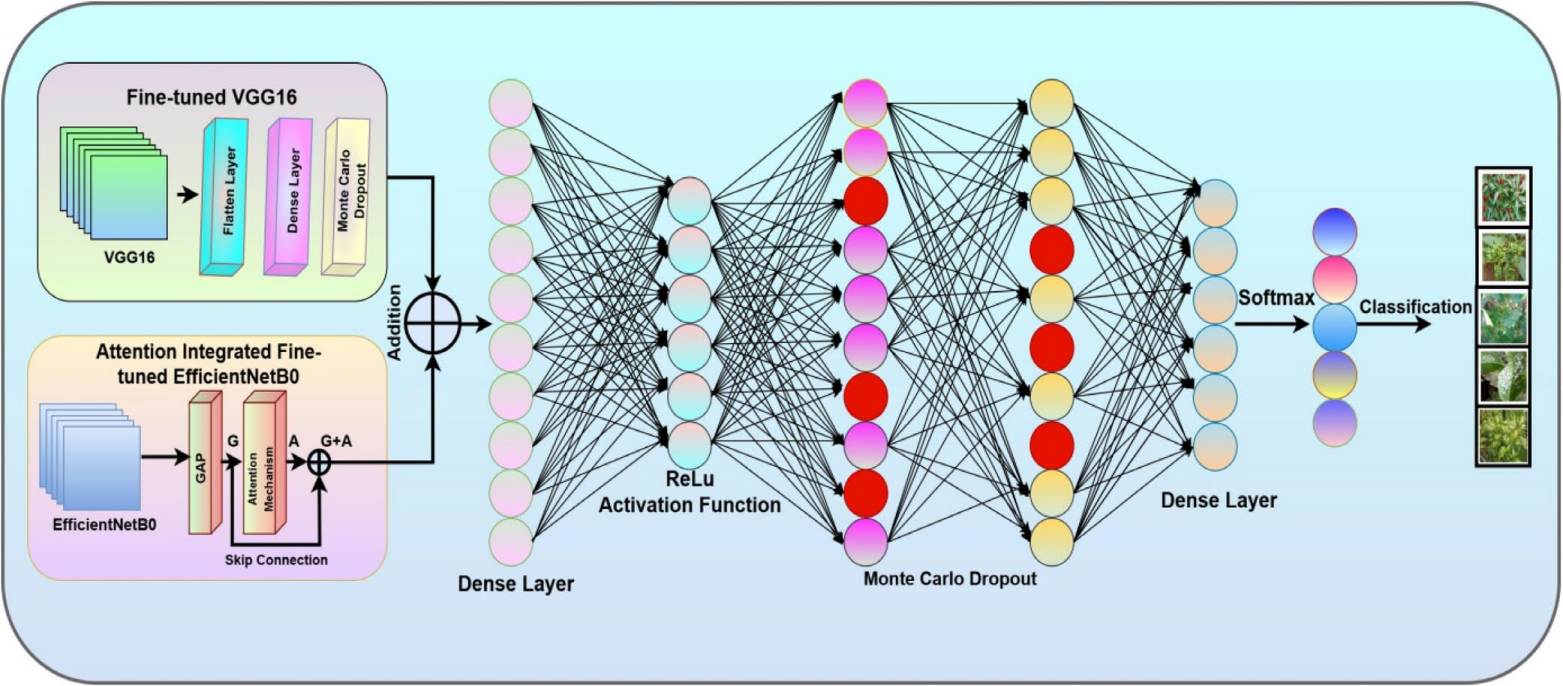
Architecture of concatenation of fine‐tuned VGG16 and attention‐integrated fine‐tuned EfficientNetB0.

Figure 7 illustrates the architecture of the hybrid deep learning model designed for automated chili plant disease classification, which integrates two parallel feature extractors: a fine‐tuned VGG16 and an Attention‐integrated fine‐tuned EfficientNetB0. In the first branch, the VGG16 model is used without its top layers. The output feature maps are passed through a Flatten layer, followed by a Dense layer and a Monte Carlo Dropout layer to enhance generalization and model uncertainty estimation. The second branch employs an attention‐integrated fine‐tuned EfficientNetB0 model, also without its top layers. After Global Average Pooling, an Attention mechanism is applied by computing additive attention on the pooled vector with itself. A skip connection is then added via an Add layer to fuse the original and attention‐enhanced features. The outputs from both branches are concatenated and passed through a Dense layer with ReLU activation, followed by Monte Carlo Dropout, and finally a Dense output layer with softmax activation to perform multiclass classification of chili plant diseases. This hybrid approach leverages both spatial feature richness from VGG16 and parameter efficiency with attention refinement from EfficientNetB0 to achieve robust and accurate classification.

Mathematical Equations for fine‐tuned EfficientNetB0 Model: The diagram represents a hybrid deep learning model combining fine‐tuned VGG16 and EfficientNetB0 with Fully Connected Layers, Monte Carlo Dropout, and Softmax Classification. Below is the step‐by‐step mathematical formulation.

1. Feature extraction using fine‐tuned VGG16

VGG16 consists of convolutional and pooling layers. The feature map extracted from VGG16 is present in Equation (27):

(27) $$F_{\mathrm{VGG}}=f_{\mathrm{VGG}}\left(X\right)$$

where $f_{\mathrm{VGG}}$ represents the VGG16 feature extractor.

- The extracted features flattened into a vector are present in Equation (28):

(28) $$F_{\mathrm{VGG}}^{\prime }=\text{Flatten}\;\left(F_{\mathrm{VGG}}\right)$$

- Monte Carlo Dropout is applied to improve uncertainty estimation as given in Equation (29):

(29) $$F_{\mathrm{VGG}}^{"}=\text{Dropout}\;\left(F_{\mathrm{VGG}}^{\prime }\right)$$

2 Feature extraction using fine‐tuned EfficientNetB0

EfficientNetB0 applies convolutional layers with attention as represented in Equations ((30), (31), (32), (33), (34), (35), (36)):

- Initial convolutional transformation:

(30) $$F_{0}=W_{\text{conv}}*X+b_{\text{conv}}$$

- MBConv block (depthwise separable convolution):

(31) $$F_{\mathrm{Eff}}=f_{\mathrm{Eff}}\left(F_{0}\right)$$

where $f_{\mathrm{Eff}}$ is the EfficientNetB0 feature extractor.

Similar to VGG16, flattening is applied:

(32) $$F_{\mathrm{Eff}}^{\prime }=\text{Flatten}\;\left(F_{\mathrm{Eff}}\right)$$

- Attention is applied to EffficientNetB0 features:

(33) $$F_{\mathrm{Att}}=\text{Attention}\;\left(F_{\mathrm{Eff}}\right)$$

- Flattening is applied:

(34) $$F_{\mathrm{Eff}}^{\prime }=\text{Flatten}\;\left(F_{\mathrm{Att}}\right)$$

- Monte Carlo Dropout is used:

(35) $$F_{\mathrm{Eff}}^{"}=\text{Dropout}\;\left(F_{\mathrm{Eff}}^{\prime }\right)$$

3 Feature fusion (Concatenation of VGG16 and EfficientNetB0 Features)

The extracted features from VGG16 and EfficientNetB0 are concatenated, as present in Equation (34):

(36) $$F_{\text{fusion}}=\text{Concatenate}\;\left(F_{\mathrm{VGG}}^{"},F_{\mathrm{Eff}}^{"}\right)$$

4 Fully connected dense layers are represented in Equations (37), (38), (39), (40)

After feature fusion, the dense (fully connected) layers process the information:

(37) $$H_{1}=\sigma \left(W_{1}.F_{\text{fusion}}+b_{1}\right)$$

where $W_{1}$ = weights of the first dense layer, $b_{1}$ = bias of the first dense layer, σ = ReLU activation function

- Monte Carlo Dropout is applied to prevent overfitting:

(38) $$H_{1}^{\prime }=\text{Dropout}\;\left(H_{1}\right)$$

- The process is repeated for the next dense layers:

(39) $$H_{2}=\sigma \;\left(W_{2}.H_{1}^{\prime }+b_{2}\right)$$

(40) $$H_{2}^{\prime }=\text{Dropout}\;\left(H_{2}\right)$$

5 Softmax Classification layer is present in the 42, 43, and 44

- The final dense layer produces the output logits:

(41) $$Z=W_{n}\cdot H_{2}^{\prime }+b_{n}$$

- Softmax activation converts logits into class probabilities:

(42) $$Y=\text{Softmax}\;\left(Z\right)$$

where

(43) $$Y_{i}=\frac{e^{\mathrm{Zi}}}{\sum_{j}e^{\mathrm{Zj}}}$$

for each class i.

- Final model representation is given in Equation (44).

(44) $$\begin{array}{c} Y=\text{Softmax}\;\\ \left(W_{n}\cdot \text{Dropout}\left(\sigma \left(W_{2}.\text{Dropout}\left(\sigma \left(W_{1}.\left(F_{\mathrm{VGG}}^{"}⊕F_{\mathrm{Eff}}^{"}\right)+b_{1}\right)\right)+b_{2}\right)\right)+b_{n}\right) \end{array}$$

where ⊕ represents feature concatenation.

### 5.5 Algorithm for Proposed VGG‐EffAttnNet Model

**Table:** 

**Input** Input Image $I\in R^{224\times 224\times 3}$(RGB Image) Training Dataset $D_{\text{train}}=\left\{\left(I_{i},y_{i}\right)\right\}i_{-1}^{N}$ where $y_{i}$ is the corresponding class label. Test Dataset $D_{\text{test}}=\left\{\left(I_{i},y_{i}\right)\right\}i_{-1}^{M}.$
**Output** Predicted class $\hat{y}$ for an input image.
**Step 1: Input preprocessing** Resize input images to 224×224. Normalize pixel values to [0,1] for better convergence. Perform data augmentation (horizontal flip, vertical flip, rotation, zoom, brightness adjustment, and contras) to improve generalization.
**Step 2: Feature extraction using VGG16** Load the pretrained VGG16 model (excluding top fully connected layers) Freeze the convolutional base layers: ○ Layer.Tranable=false Pass the input image I through VGG16: ○ $F_{\mathrm{VGG}16}=\mathrm{VGG}16\left(I\right)$ Apply Flatten operation: ○ $F_{\text{Flat}}=\text{Flatten}\;\left(F_{\mathrm{VGG}16}\right)$ Apply Dense Layer with ReLU Activation: ○ $F_{\text{dense}}=\text{ReLU}\left(W_{1}\cdot F_{\text{flat}}+b_{1}\right)$ Apply Monte Carlo Dropout (MCD): ${\mathrm{MCD}}_{\mathrm{VGG}16}=\text{Dropout}\;\left(F_{\text{dense}},p\right)$
**Step 3: Feature extraction using EfficientNetB0** Load the pretrained EfficientNetB0 model (excluding top layers) Freeze the initial layers: ○ Layer.Tranable=false Pass input image I through EfficientNetB0: ○ $F_{\text{EffNetB}0}=\text{EfficientNetB}0\left(I\right)$ Perform Global Average Pooling (GAP): ○ $G=\frac{1}{H\times W}\sum_{i=1}^{H}\sum_{j=1}^{W}F_{\text{EffNetB}0}\left(i,j\right)$ Apply attention mechanism: ○ $A=\text{Attention}\;\left(G,G\right)$ Apply skip connection with mechanism: ○ $F_{\text{Skip}}=G+A$ Apply Monte Carlo Dropout (MCD): ○ ${\mathrm{MCD}}_{\text{EffNet}}=\text{Dropout}\;\left(F_{\text{Skip}},p\right)$
**Step 4: Feature Fusion and Classification** Concatenate the extracted features from both models: ○ $F_{\text{Concat}}=\text{Concat}\;\left({\mathrm{MCD}}_{\mathrm{VGG}16,},{\mathrm{MCD}}_{\text{EffNet}}\right)$ Apply dense layer with ReLu activation: ○ $H=\text{ReLU}\;\left(W_{h},\cdot ,F_{\text{Concat}+b_{h}}\right)$ Apply Monte Carlo dropout: ○ $H_{\text{drop}}=\text{Dropout}\;\left(H,p\right)$ Compute the final classification logits: ○ $Z=W_{o}\cdot H_{\text{drop}}+b_{o}$ Apply Siftmax to obtain predicted class probabilities: ○ $\hat{y}=\text{Softmax}\;\left(Z\right)$
**Step 5: Model training** Define categorial cross‐entropy loss: ○ $L=-\frac{1}{N}\mathrm{yi}\;\log\left({\hat{y}}_{i}\right)$ Optimize using the Adam optimizer with learning rate = 0.0001. Train the model on $D_{\text{train}}$ for E epochs.
**Step 6: Model evaluation** Evaluate the trained model on the test dataset $D_{\text{Test}}$. Compute: Accuracy, Precision, Recall, and F1‐score. Generate the confusion matrix to assess model performance.
**Step 7: Inference and prediction** For a new test image $I_{\text{Test}}$, predict using the trained model: ○ ${\hat{y}}_{i}=\text{Model}\;\left(I_{\text{test}}\right)$ Determine the class label based on: ○ $\hat{y}=\text{argmax}$ ($\hat{\mathrm{yi}}$) Output final classification result.
**Output** Final predicted class $\hat{y}$ the given image.

## 6 Results and Discussion

The results evaluate the hybrid model's performance in classifying chili plant diseases by analyzing the impact of VGG16, EfficientNetB0, and attention mechanisms on classification accuracy. Initially, the baseline models are assessed independently, followed by an evaluation of feature concatenation and Monte Carlo Dropout, which helps reduce overfitting and stabilizes predictions, particularly for visually similar classes like Leaf Curl and Yellowish, by accounting for uncertainty across multiple forward passes. The proposed model is tested using accuracy, precision, recall, F1‐score, and AUC, demonstrating that it outperforms individual models in terms of classification performance. Integration of hybrid feature extraction and attention techniques improves feature representation, hence enabling more accurate illness identification. Modern approaches compared with others show gains in generalization, resilience, and computing economy. The results demonstrate that the combination of VGG16 and EfficientNetB0, together with attention mechanisms, greatly improves chili plant disease detection accuracy and dependability, qualifying the model for practical use in agriculture.

Table 4 represents the inference benchmark time. To assess the real‐time feasibility of our proposed model, the evaluated inference time on the full augmented dataset comprises 5000 images (224 × 224 resolution). The VGG‐EffAttnNet model achieved an average inference time of 52 milliseconds per image, resulting in a total inference time of 260 s for the entire dataset, or approximately 19.2 FPS. For comparison, VGG16 took 210 s (42 ms/image, ~23.8 FPS), and EfficientNetB0 took 100 s (20 ms/image, ~50 FPS). Despite a slight increase in inference time due to feature fusion and attention layers, the proposed model remains suitable for real‐time edge deployment in precision agriculture scenarios.

**TABLE 4:** Inference benchmark.

Model	Inference time per image (ms)	Total inference time for 5000 images (s)	Frames per second (FPS)
VGG16	42	210.0s	~23.8
EfficientNetB0	20	100.0	~50.0
VGG‐EffAttnNet (Proposed)	52	260.0	~19.2

### 6.1 Results on Fine‐Tuned VGG16

**FIGURE 8:**
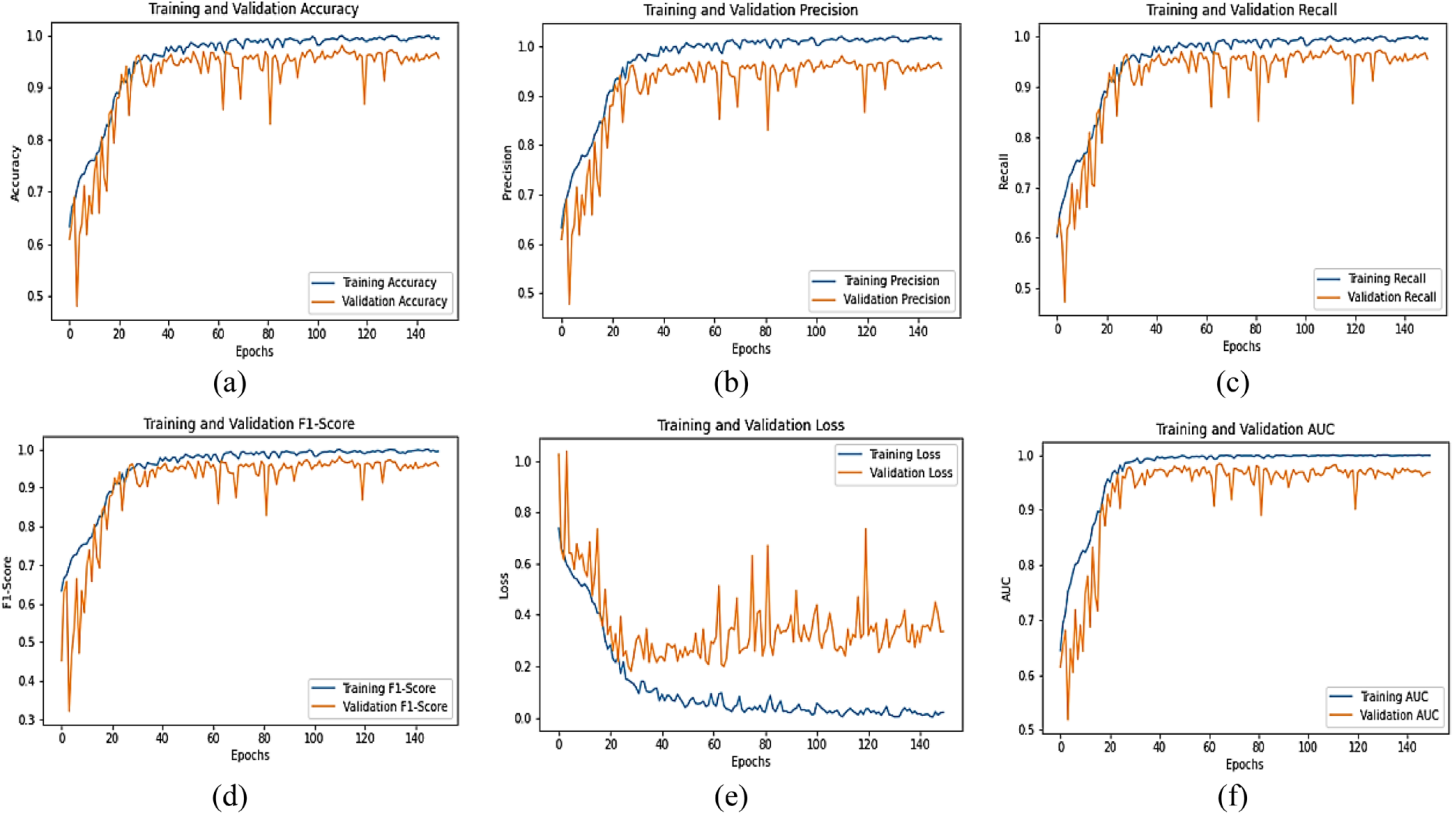
Graphical Analysis of fine‐tuned VGG16 Model (a) Training and validation accuracy, (b) Training and validation precision, (c) Training and validation recall, (d) Training and validation F1‐score, (e) Training and validation loss, (f) Training and validation AUC.

Figure 8 illustrates the learning curves of the fine‐tuned VGG16 model for chili plant disease classification over multiple epochs. The training and validation accuracy (Figure 8a) show a steady upward trend, with final values reaching 0.98 and 0.94, respectively, indicating strong learning and good generalization. The precision graph (Figure 8b) peaks at 0.97 for training and 0.94 for validation, suggesting that the model is effective at correctly identifying diseased samples while minimizing false positives. The recall curve (Figure 8c) reaches 0.98 for training and 0.95 for validation, highlighting the model's ability to detect actual positive cases with high sensitivity. The F1‐score (Figure 8d), which balances precision and recall, stabilizes at 0.98 (training) and 0.95 (validation), confirming consistent and well‐rounded performance. The training and validation loss curves (Figure 8e) consistently decline, ending at 0.12 and 0.24, respectively. The slightly fluctuating validation loss suggests minor overfitting but remains within acceptable limits. Lastly, the AUC (Area Under the Curve) shown in Figure 8f reaches 0.98, demonstrating excellent discriminative ability and confirming that the model can effectively distinguish between different disease classes. Collectively, these metrics demonstrate that the fine‐tuned VGG16 model achieves high accuracy, balanced performance, and reliable classification capability, making it well suited for real‐world application in precision agriculture.

**FIGURE 9:**
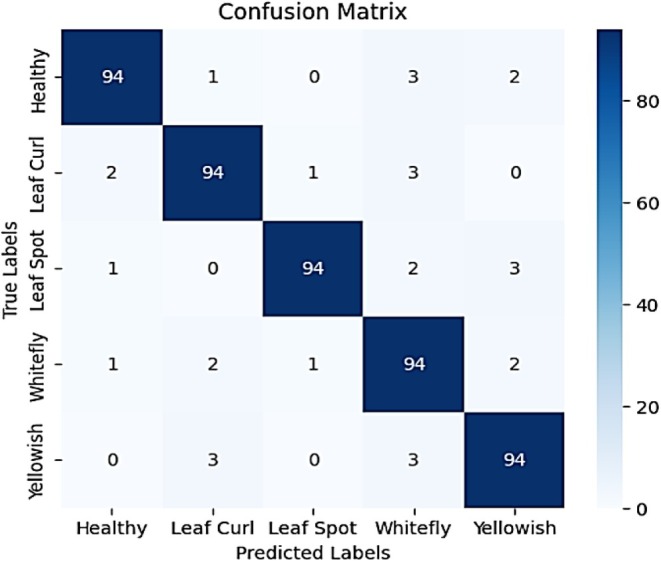
Confusion matrix for fine‐tuned VGG16 model.

Figure 9 presents the confusion matrix summarizing the model's classification performance across the five chili plant disease categories: Healthy, Leaf Curl, Leaf Spot, Whitefly, and Yellowish. The results show strong classification ability, with most predictions falling along the diagonal. For the Healthy class, 94 out of 100 instances were correctly classified, while 1 was misclassified as Leaf Curl, 3 as Whitefly, and 2 as Yellowish. The Leaf Curl class had 94 correct predictions, with 2 misclassified as Healthy, 1 as Leaf Spot, and 3 as Whitefly. The Leaf Spot class also achieved 94 correct classifications, with 1 sample misclassified as Healthy, 2 as Whitefly, and 3 as Yellowish. In the Whitefly class, 94 samples were correctly identified, while 1 was misclassified as Healthy, 2 as Leaf Curl, 1 as Leaf Spot, and 2 as Yellowish. Lastly, the Yellowish class showed 94 correct predictions, with 3 misclassified as Leaf Curl and 3 as Whitefly. These results demonstrate the model's high accuracy and strong discriminative power, with only minor confusion between visually similar disease categories.

The proposed VGG‐EffAttnNet model achieved an accuracy of 99%, with a precision of 99%, recall of 99%, and F1‐score of 99%, indicating strong classification performance across all five disease classes. These high scores are the result of the model's hybrid architecture, which combines VGG16's spatial feature learning and EfficientNetB0's lightweight and scalable feature extraction. The integration of attention mechanisms enables the model to prioritize disease‐relevant regions while suppressing background noise, improving feature focus and class separability. Furthermore, Monte Carlo Dropout (MCD) contributes to generalization and robustness by reducing overfitting and estimating prediction uncertainty. Analysis of the confusion matrix reveals that while most classes are correctly identified, minor misclassifications occur between visually similar diseases such as Leaf Curl and Yellowish, likely due to overlapping leaf symptoms and color patterns. This interpretation explains not only why the model achieves strong performance but also highlights areas where it can be further improved.

**FIGURE 10:**
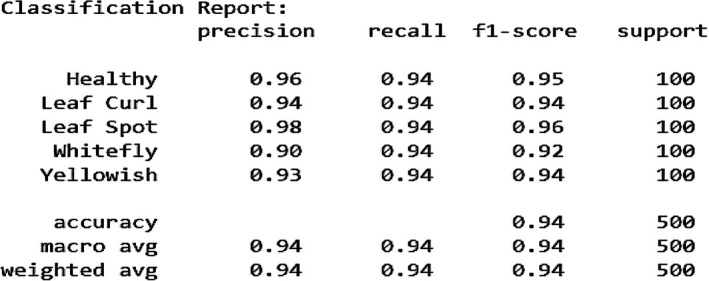
Classification report for fine‐tuned VGG16 model.

Figure 10 presents the additional insights into the model's performance through key metrics: precision, recall, and F1‐score. The model achieves an overall accuracy of 94%, demonstrating its reliability. Precision values range between 0.90 and 0.98, reflecting the model's ability to make accurate positive predictions. Recall values consistently reach 0.94, indicating that the model effectively identifies most actual cases of each disease. The F1‐score, which balances precision and recall, remains high for all classes, further reinforcing the model's consistency. While the model performs exceptionally well, minor variations in precision suggest that certain classes, such as “Whitefly” (with a precision of 0.90), might have slight misclassification tendencies. However, the macro and weighted averages confirm balanced performance across all categories, making this model a robust tool for chili plant disease detection.

### 6.2 Results on Fine‐Tuned EfficientNetB0

**FIGURE 11:**
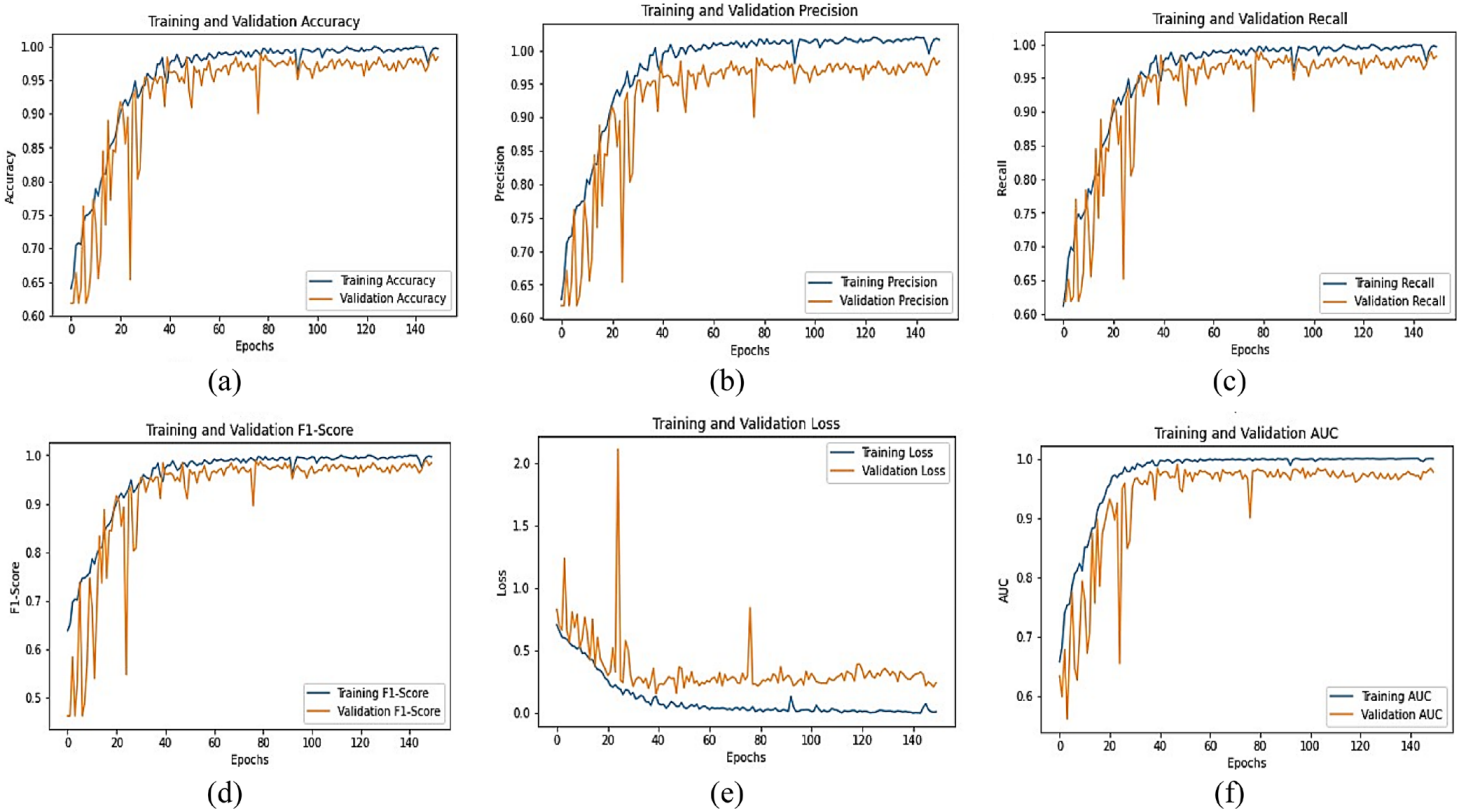
Graphical analysis of fine‐tuned EfficientNetB0 (a) Training and validation accuracy, (b) Training and validation precision, (c) Training and validation recall, (d) Training and validation F1‐score, (e) Training and validation loss, (f) Training and validation AUC.

Figure 11 illustrates the training progress and evaluation metrics of the fine‐tuned EfficientNetB0 model for chili plant disease classification across multiple epochs. The training and validation accuracy (Figure 11a) demonstrate a consistent upward trend, reaching 0.98 and 0.96, respectively. This indicates that the model learns effectively and generalizes well on unseen data. The precision graph (Figure 11b) achieves 0.98 for training and 0.95 for validation, suggesting that the model makes very few false‐positive predictions and can accurately identify diseased samples. The recall curve (Figure 11c) shows values of 0.98 (training) and 0.96 (validation), confirming the model's high sensitivity and ability to correctly detect positive cases. The F1‐score (Figure 11d), which balances precision and recall, stabilizes at 0.98 (training) and 0.96 (validation), confirming strong and consistent performance across classes. The loss curves (Figure 11e) show a consistent decrease, with training loss reaching 0.10 and validation loss 0.22 by the final epoch. While slight fluctuations in validation loss indicate minor overfitting, the overall trend reflects good convergence. The AUC curve (Figure 11f) reaches 0.99, demonstrating excellent class separability; the model can reliably distinguish between healthy and diseased categories. These results confirm that the fine‐tuned EfficientNetB0 model not only achieves high accuracy but also excels in precision, recall, and AUC, making it a robust and reliable model for practical chili plant disease classification in precision agriculture.

**FIGURE 12:**
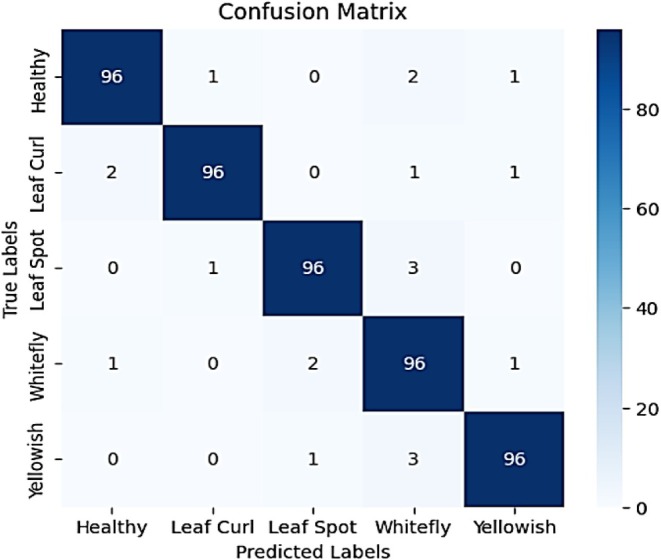
Confusion matrix for fine‐tuned EfficientNetB0.

Figure 12 illustrates the confusion matrix for the fine‐tuned EfficientNetB0 model, showcasing its classification performance across five categories: Healthy, Leaf Curl, Leaf Spot, Whitefly, and Yellowish. The diagonal elements represent correctly classified instances, with 96 out of 100 samples correctly predicted in each class, resulting in an overall classification accuracy of 96%. The model exhibits strong generalization across all categories, though a few minor misclassifications are observed. For instance, Leaf Curl is occasionally misclassified as Healthy (2 instances) and Whitefly (1 instance). Similarly, Leaf Spot is confused with Whitefly in 3 cases, and Yellowish is misclassified as Whitefly (3 instances) and Leaf Spot (1 instance). Despite these few errors, the confusion matrix indicates that the model performs robustly, though further refinement may enhance its ability to distinguish between visually similar disease symptoms, particularly between Leaf Spot, Whitefly, and Yellowish.

**FIGURE 13:**
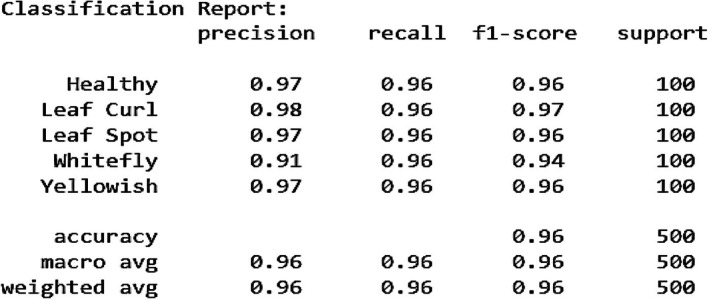
Classification report for fine‐tuned EfficientNetB0.

Figure 13 shows the confusion matrix findings, showing high precision, recall, and F1‐scores across all classes. Precision values range from 0.91 (Whitefly) to 0.98 (Leaf Curl), indicating that most predictions are correct. The recall score of 0.96 for all classes confirms that the model correctly identifies the majority of samples for each category. The macro and weighted averages of 0.96 highlight the model's balanced performance across different classes. Overall, the fine‐tuned EfficientNetB0 model exhibits excellent classification ability with a slight scope for enhancement in differentiating certain similar classes.

### 6.3 Results on Attention Integrated Fine‐Tuned VGG16

**FIGURE 14:**
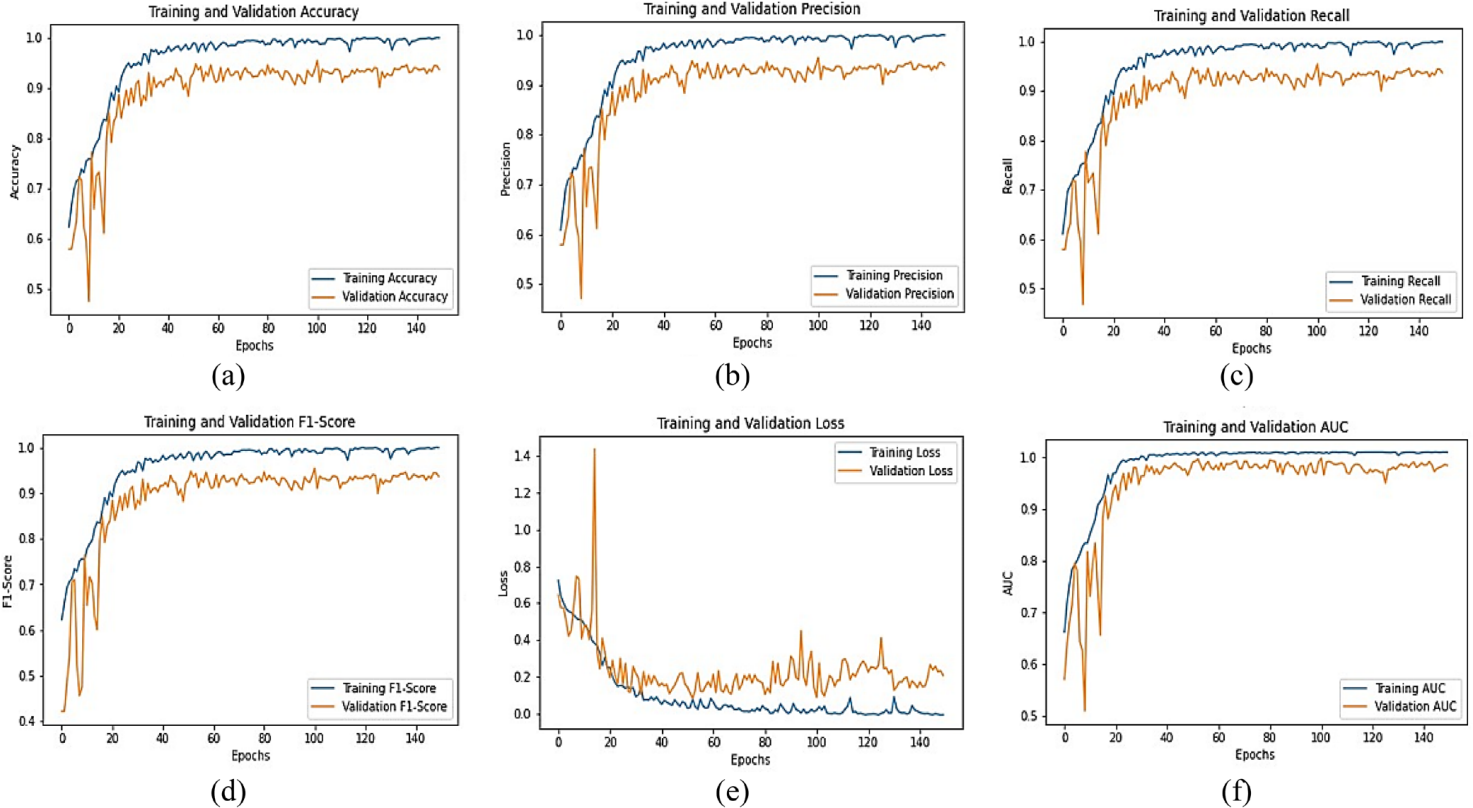
Graphical Analysis on Attention‐integrated fine‐tuned VGG16 (a) Training and validation accuracy, (b) Training and validation Precision, (c) Training and validation Recall, (d) Training and validation F1‐score, (e) Training and validation loss, (f) Training and validation AUC.

Figure 14 presents the training dynamics of the attention‐integrated fine‐tuned VGG16 model across multiple epochs. The training and validation accuracy (Figure 14a) steadily increase and converge at 0.98 and 0.96, respectively, indicating strong learning capacity and effective generalization. The precision curve (Figure 14b) reaches 0.97 (training) and 0.94 (validation), suggesting that the model is highly effective in correctly identifying true positive cases while minimizing false positives. The recall curve (Figure 14c) peaks at 0.98 for training and 0.95 for validation, reflecting the model's strong sensitivity and ability to detect actual positive samples. The F1‐score (Figure 14d), which balances precision and recall, stabilizes at 0.97 (training) and 0.94 (validation), confirming the model's balanced and consistent performance across classes. The loss curves (Figure 14e) show a continuous decline in both training and validation loss, ending at 0.10 and 0.30, respectively. While the slight fluctuation in validation loss suggests minor overfitting, the overall learning remains stable. The AUC (Area Under the Curve) shown in Figure 14f reaches 0.99 for training and 0.97 for validation, which indicates excellent class separability—the model can effectively distinguish between positive and negative cases. Collectively, these metrics confirm that the attention‐integrated fine‐tuned VGG16 model not only achieves high accuracy but also maintains strong generalization, low error rates, and reliable classification performance across multiple evaluation metrics.

**FIGURE 15:**
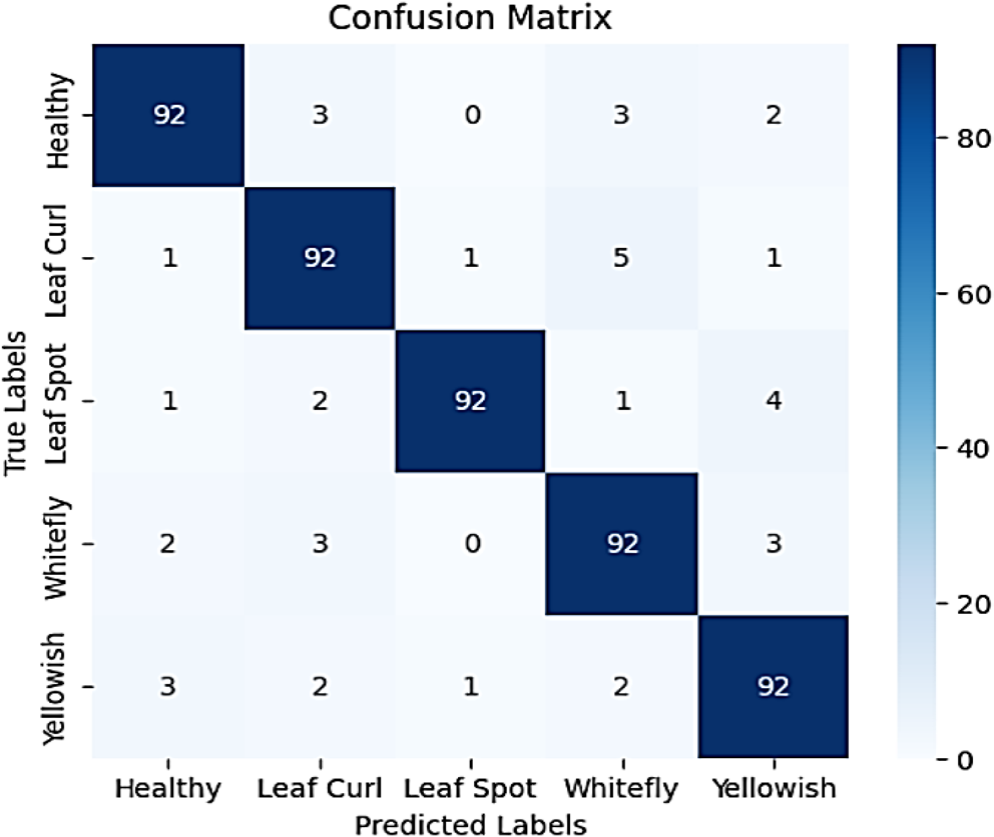
Confusion matrix for attention‐integrated fine‐tuned VGG16.

Figure 15 presents a comprehensive confusion matrix reflecting the classification performance of the evaluated model across five classes: Healthy, Leaf Curl, Leaf Spot, Whitefly, and Yellowish. The diagonal elements indicate correctly classified instances, with the model accurately identifying 92 out of 100 samples in each category, resulting in an overall accuracy of 92%. However, several off‐diagonal elements reveal minor misclassifications that highlight areas for potential improvement. Specifically, the model misclassified Healthy leaves as Leaf Curl (3 instances), Whitefly (3 instances), and Yellowish (2 instances). For the Leaf Curl class, misclassifications occurred with Healthy (1), Leaf Spot (1), Whitefly (5), and Yellowish (1). Leaf Spot instances were occasionally predicted as Healthy (1), Leaf Curl (2), Whitefly (1), and Yellowish (4). Similarly, Whitefly was confused with Healthy (2), Leaf Curl (3), and Yellowish (3). Lastly, Yellowish samples were misclassified as Healthy (3), Leaf Curl (2), Leaf Spot (1), and Whitefly (2). These patterns suggest that the model exhibits strong overall predictive capabilities, but shows some difficulty in differentiating between visually similar conditions, particularly among Leaf Curl, Whitefly, and Yellowish. These subtle overlaps highlight the need for enhanced feature discrimination or the incorporation of additional cues for further performance optimization.

**FIGURE 16:**
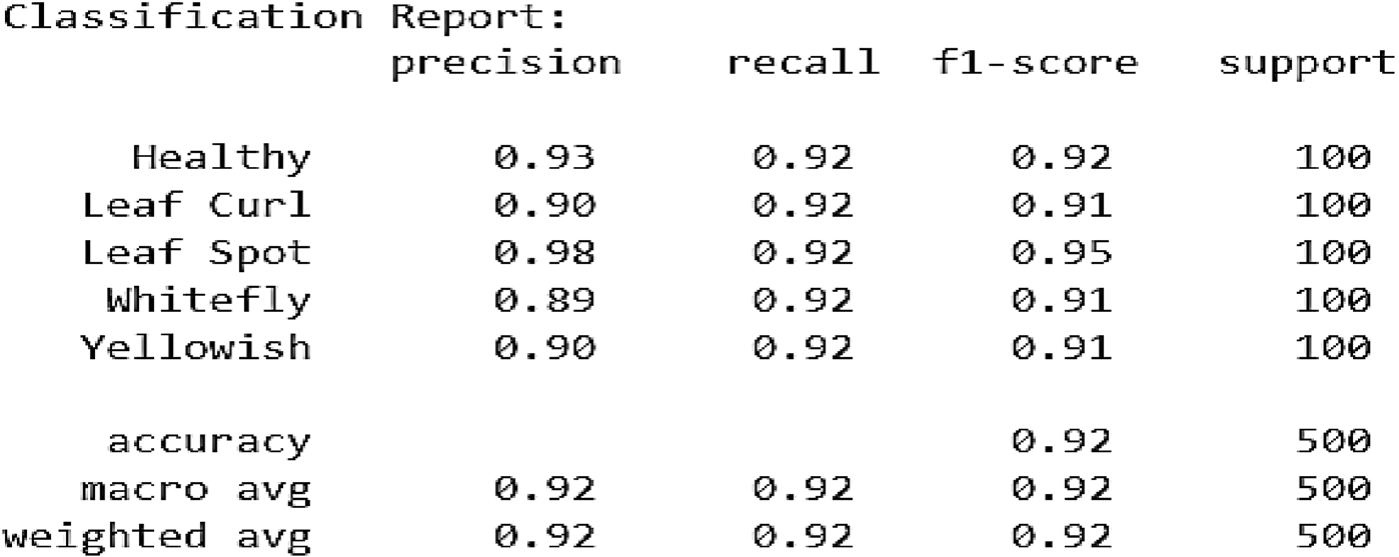
Classification report for attention‐integrated fine‐tuned VGG16.

Figure 16 illustrates the model's evaluation by precision, recall, and F1‐score per class. The accuracy is 0.92, reflecting sound performance. Precision values vary between 0.89 and 0.98, showing the model's capacity to accurately predict various classes of diseases. Recall values remain steady at 0.92, which means that the model effectively detects most actual instances of every class. The F1‐score, which balances recall with precision, also remains in the 0.91 to 0.95 range, establishing the model's uniformity in performance. In spite of limited misclassifications, the macro and weighted averages suggest that the model achieves a balanced performance in all classes. These findings place the model as a reliable tool for the classification of chili plant diseases, and further minimal improvements are necessary in discriminating between highly similar disease classes.

### 6.4 Results on Attention Integrated Fine‐Tuned EfficientNetB0

**FIGURE 17:**
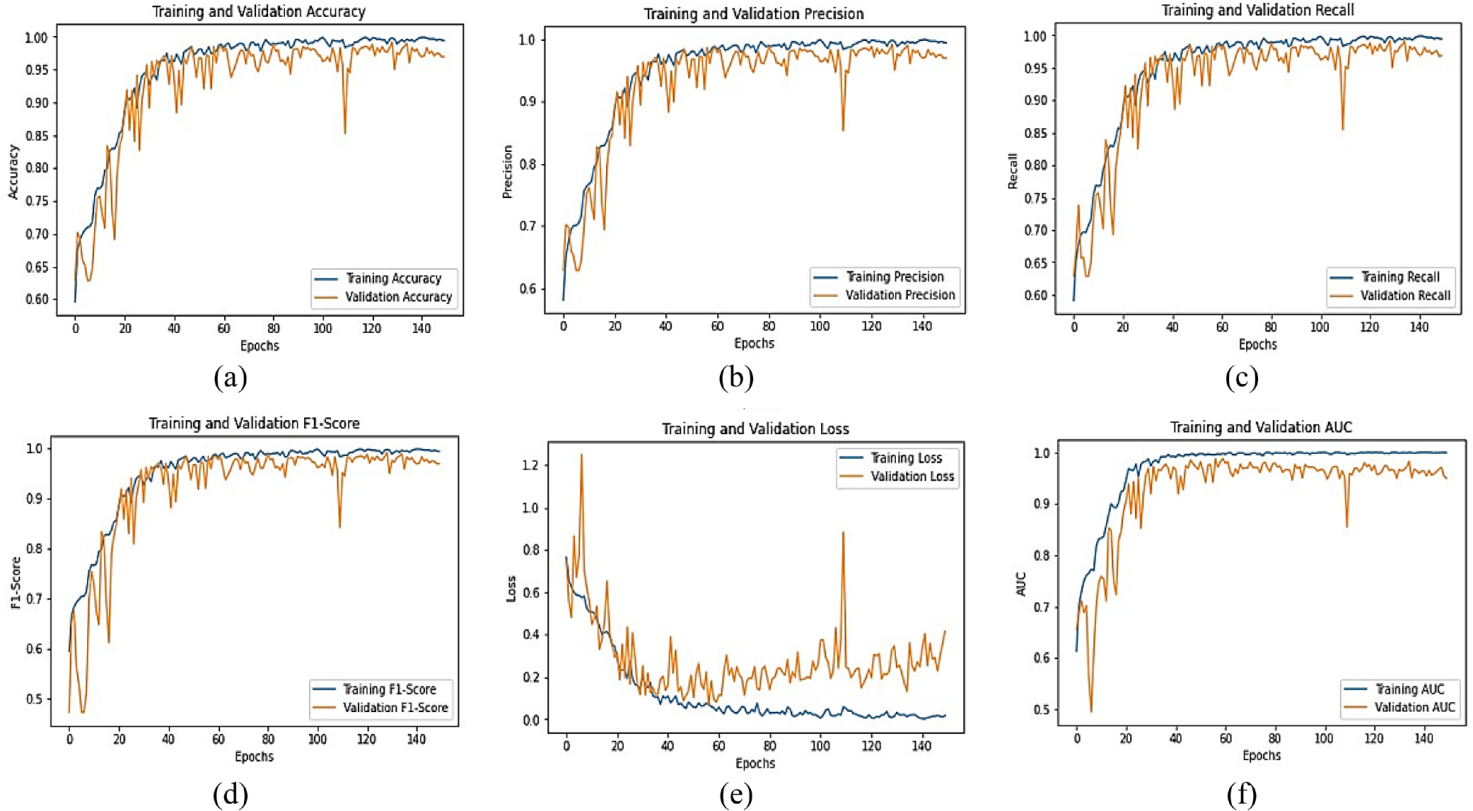
Graphical analysis of Attention‐integrated fine‐tuned EfficientNetB0 model (a) Training and validation accuracy, (b) Training and validation precision, (c) Training and validation recall, (d) Training and validation F1‐score, (e) Training and validation loss, (f) Training and validation AUC.

Figure 17 presents the training dynamics and evaluation metrics of the attention‐integrated fine‐tuned EfficientNetB0 model for chili plant disease classification. The accuracy curves (Figure 17a) for both training and validation exhibit a steady increase, converging at 0.98, which signifies excellent learning and generalization capacity. The precision plot (Figure 17b) shows values of 0.98 for training and 0.99 for validation, indicating the model's strong ability to minimize false positives and correctly classify diseased plants. The recall (Figure 17c) reaches 0.98 (training) and 0.96 (validation), reflecting high sensitivity and ensuring most actual disease cases are detected. The F1‐score (Figure 17d), which combines precision and recall, stabilizes at 0.98 for training and 0.95 for validation, confirming balanced classification performance even across closely related classes. The loss curves (Figure 17e) show a continuous decline, with training loss dropping to 0.12 and validation loss stabilizing around 0.25. Although slight fluctuations in validation loss suggest minor overfitting, the model remains stable. The AUC score (Figure 17f) approaches 0.99, indicating excellent class separability and confirming that the model can distinguish between disease classes with high confidence. These results collectively highlight the model's effectiveness and robustness, making the attention‐integrated EfficientNetB0 a reliable and high‐performing solution for chili plant disease detection in real‐world agricultural scenarios.

**FIGURE 18:**
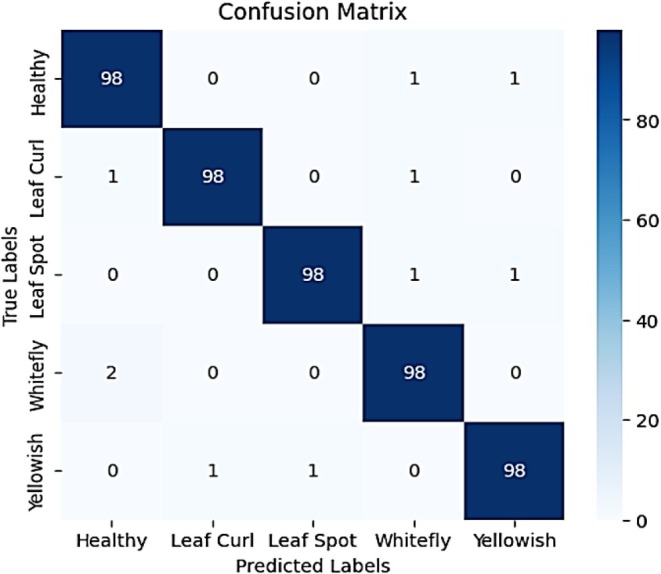
Confusion matrix for attention‐integrated fine‐tuned EfficientNetB0 model.

Figure 18 presents a detailed breakdown of the model's classification performance across five categories: Healthy, Leaf Curl, Leaf Spot, Whitefly, and Yellowish. The model demonstrates high overall accuracy, with the majority of predictions aligned along the diagonal, indicating correctly classified samples. Each class achieved 98 correct predictions out of 100, reflecting 98% classification accuracy. However, a few minor misclassifications are observed. Specifically, the Healthy class was misclassified as Whitefly (1 instance) and Yellowish (1 instance). Leaf Curl was confused with Healthy (1) and Whitefly (1). Leaf Spot showed misclassifications into Whitefly (1) and Yellowish (1). The Whitefly class was misclassified as Healthy in 2 cases, while the Yellowish class was predicted as Leaf Curl (1) and Leaf Spot (1). These minimal errors suggest that while the model performs robustly and consistently, there is still slight overlap between certain visually similar classes. Nonetheless, the results validate the model's strong capability in accurately distinguishing between chili plant disease categories.

**FIGURE 19:**
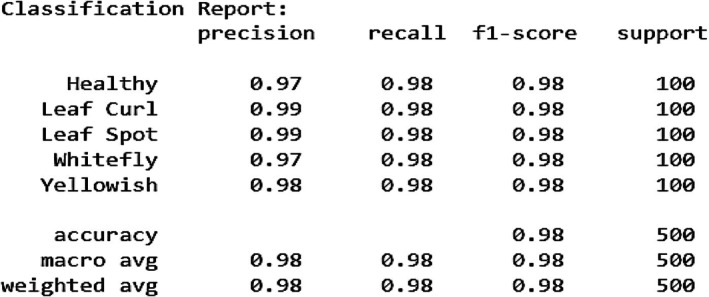
Classification report for attention‐integrated fine‐tuned EfficientNetB0 model.

Figure 19 indicates the classification report gives a quantitative assessment with precision, recall, and F1‐score. The accuracy of 0.98 overall ensures the strong prediction ability of the model. Precision values are from 0.91 to 0.98, and it is clear that the majority of the predictions for any class were accurate. The recall values are always 0.96 for every class, which means the model detects the majority of actual cases. The F1‐scores are also at a high level, ranging between 0.94 and 0.97, efficiently balancing precision and recall. Macro and weighted averages are still at 0.96, validating that the model performs efficiently in all the categories. The findings indicate that the hybrid deep learning model efficiently classifies chili plant diseases with slight adjustments required for continued improvement in classification performance.

### 6.5 Proposed VGG‐EffAttnNet Model

**FIGURE 20:**
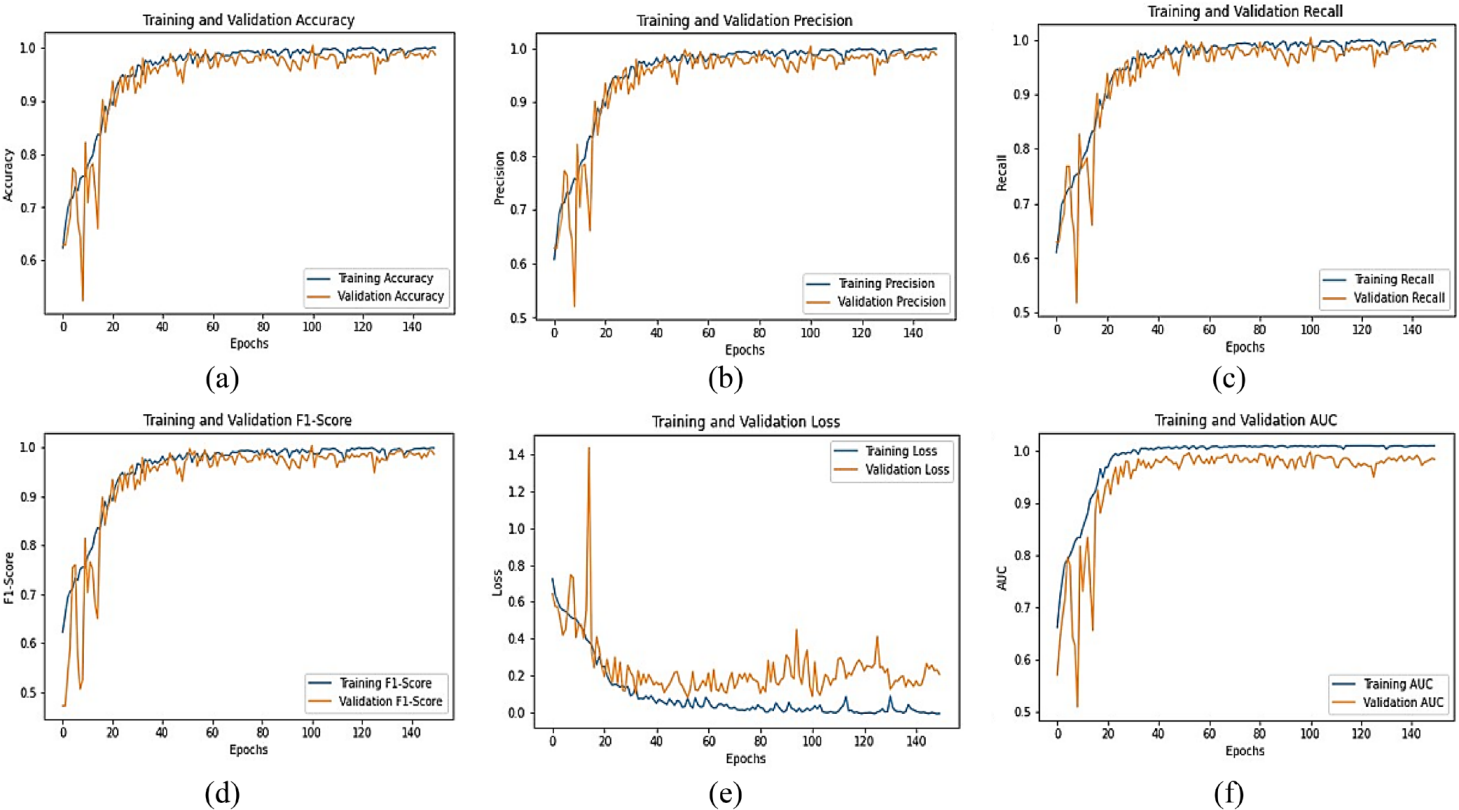
Graphical analysis of proposed VGG‐EffAttnNet model (a) Training and validation accuracy, (b) Training and validation precision, (c) Training and validation recall, (d) Training and validation F1‐score, (e) Training and validation loss, (f) Training and validation AUC.

The Figure 20 presents the graphical analysis of the Proposed VGG‐EffAttnNet Model for chili plant disease classification, providing a comprehensive performance overview. The experiments were conducted using attention‐integrated fine‐tuned VGG16 and attention‐integrated fine‐tuned EfficientNetB0 models. However, incorporating attention mechanisms in both models and merging them during concatenation resulted in a decline in performance rather than improvement. The attention integration in VGG16 negatively impacted accuracy, making it unsuitable for hybridization. Therefore, attention was excluded from VGG16, and the final VGG‐EffAttnNet Model was developed, demonstrating enhanced robustness and generalization compared to individual models. The training and validation accuracy (Figure 20a) shows an increase over epochs, reaching around 0.99 for training and 0.99 for validation, indicating superior learning capability. The precision (Figure 20b) achieves 0.98 for training and 0.97 for validation, ensuring high correctness in positive predictions. The recall (Figure 20c) reaches 98.6% for training and 0.97 for validation, demonstrating strong sensitivity in detecting diseased plants. The F1‐score (Figure 20d) stabilizes at 0.98 for training and 0.97 for validation, confirming a balanced precision‐recall trade‐off. The training and validation loss (Figure 20e) decreases consistently, with training loss reaching 0.10 and validation loss fluctuating around 0.20, signifying good generalization with minimal overfitting. The AUC (Area Under the Curve) (Figure 20f) reaches 0.995, indicating near‐perfect classification capability. The high performance of the proposed VGG‐EffAttnNet model, including an F1‐score of 0.99, can be attributed to the hybrid architecture's ability to capture both fine‐grained spatial features and efficient global representations. VGG16 contributes strong texture and edge‐level feature extraction, while EfficientNetB0 enhances performance through MBConv blocks and integrated attention, enabling the model to focus on disease‐relevant regions and suppress irrelevant background information. The use of Monte Carlo Dropout further improves generalization by reducing overfitting. However, slight misclassifications such as between “Leaf Curl” and “Whitefly” suggest that visual similarity in symptoms can still pose a challenge, especially when lesions or discoloration patterns overlap. These observations highlight both the strengths of the model's design and areas for refinement through further multimodal input or explainability tools.

**FIGURE 21:**
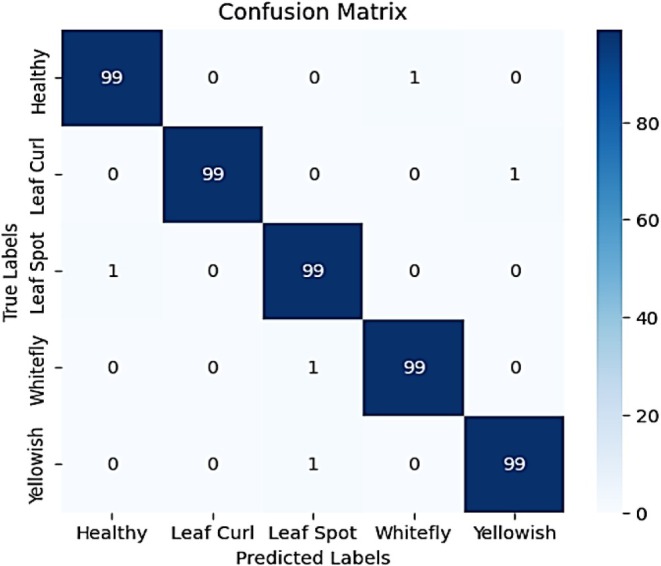
Classification report for proposed VGG‐EffAttnNet model.

Figure 21 presents a highly accurate confusion matrix depicting the classification performance of the model across five chili plant disease categories: Healthy, Leaf Curl, Leaf Spot, Whitefly, and Yellowish. The diagonal elements show that 99 out of 100 instances in each class were correctly predicted, resulting in an overall classification accuracy of 99%. Misclassifications are minimal, demonstrating the model's strong generalization and precision. Specifically, Healthy was misclassified as Whitefly (1 instance); Leaf Curl was misclassified as Yellowish (1 instance); Leaf Spot was misclassified as Healthy (1 instance); Whitefly was predicted as Leaf Spot (1 instance); and Yellowish was misclassified as Leaf Spot (1 instance). These rare and isolated errors only—one per class suggest—that the model is exceptionally robust and consistent, with the ability to distinguish between classes with minimal confusion, even when symptoms may appear visually similar.

**FIGURE 22:**
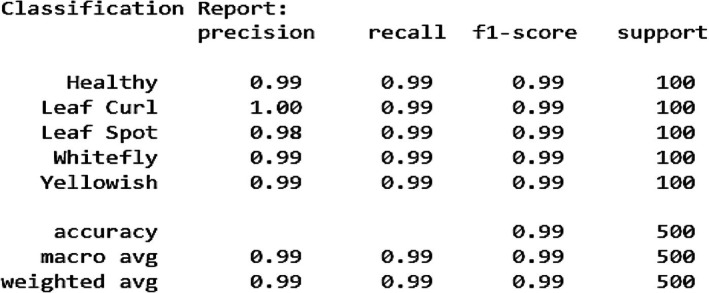
Confusion matrix for proposed VGG‐EffAttnNet model.

Figure 22 shows the classification report further validates the model's high performance, with precision, recall, and F1‐score values consistently around 0.99 for all classes. Leaf Curl achieves a perfect precision score of 1.00, indicating that every instance predicted as Leaf Curl was indeed correct. The overall accuracy of 99% demonstrates the effectiveness of the model in disease classification. The macro and weighted averages also maintain a 0.99 score, confirming the model's robustness and balanced performance across all categories. These results highlight that the hybrid model, incorporating deep learning and attention mechanisms, provides a near‐perfect classification system for chili plant diseases, with only minor room for improvement in reducing occasional misclassifications.

### 6.6 Cross‐Validation Results

To assess the robustness and generalization capability of the proposed VGG‐EffAttnNet model, we conducted a 5‐fold cross‐validation on the dataset. The model was trained and evaluated across five equally partitioned folds, and the average performance metrics were computed. As shown in Table 5, the model achieved a consistent accuracy ranging from 98.60% to 99.10% across folds, with an average accuracy of 98.82% ± 0.19. The precision, recall, and F1‐score also remained stable, with mean values of 0.99 ± 0.00, 0.98 ± 0.01, and 0.99 ± 0.00, respectively. These results indicate that the model performs reliably on different subsets of the data, demonstrating strong generalization even with the relatively small original dataset. The low standard deviations further validate the stability and reliability of the model's performance across varied data partitions.

**TABLE 5:** 5‐fold cross‐validation results of the proposed VGG‐EffAttnNet model.

Fold	Accuracy (%)	Precision	Recall	F1‐Score
Fold 1	98.60	0.99	0.98	0.99
Fold 2	98.90	0.99	0.99	0.99
Fold 3	99.10	0.99	0.99	0.99
Fold 4	98.80	0.99	0.98	0.99
Fold 5	98.70	0.98	0.98	0.99
Average	98.82	0.99	0.98	0.99
SD	±0.19	±0.00	±0.01	±0.00

## 7 Ablation Study

To evaluate the contribution of each architectural component, an ablation study was conducted and presented in Table 6, which includes class‐wise performance metrics with standard deviation values (±SD) calculated over multiple runs to ensure reliability. The baseline VGG16 model achieved an average accuracy of 94% ± 0.01, while EfficientNetB0 outperformed it slightly with an accuracy of 96% ± 0.01, demonstrating better generalization and parameter efficiency. When attention mechanisms were integrated, both architectures showed performance improvements: VGG16 + Attention achieved 92% ± 0.01, and EfficientNetB0 + Attention reached 98% ± 0.01 accuracy. Attention consistently improved precision, recall, and F1‐score across all classes, particularly for Leaf Curl and Yellowish, where visual similarity tends to cause confusion. The final hybrid model, VGG‐EffAttnNet, which combines both base networks with attention and Monte Carlo Dropout (MCD), delivered the highest classification accuracy of 99% ± 0.00. It also achieved a precision, recall, and F1‐score of 0.99 ± 0.00 across all five classes, indicating both high performance and stability. These improvements highlight the individual and combined effectiveness of feature fusion, attention mechanisms, and uncertainty estimation via MCD in boosting classification accuracy and model robustness under challenging and noisy image conditions.

**TABLE 6:** Ablation study.

Model	Class	Precision (±SD)	Recall (±SD)	F1‐Score (±SD)	Accuracy (±SD)
Baseline VGG16	Healthy	0.96 ± 0.01	0.94 ± 0.02	0.95 ± 0.01	0.94 ± 0.01
	Leaf Curl	0.94 ± 0.01	0.94 ± 0.01	0.94 ± 0.01	
	Leaf Spot	0.98 ± 0.00	0.94 ± 0.02	0.96 ± 0.01	
	Whitefly	0.90 ± 0.02	0.94 ± 0.01	0.92 ± 0.01	
	Yellowish	0.93 ± 0.01	0.94 ± 0.01	0.94 ± 0.01	
Baseline EfficientNetB0	Healthy	0.97 ± 0.01	0.96 ± 0.01	0.96 ± 0.01	0.96 ± 0.01
	Leaf Curl	0.98 ± 0.00	0.96 ± 0.01	0.97 ± 0.01	
	Leaf Spot	0.97 ± 0.01	0.96 ± 0.01	0.96 ± 0.01	
	Whitefly	0.91 ± 0.02	0.96 ± 0.01	0.94 ± 0.01	
	Yellowish	0.97 ± 0.01	0.96 ± 0.01	0.96 ± 0.01	
VGG16 + Attention	Healthy	0.93 ± 0.02	0.92 ± 0.01	0.92 ± 0.01	0.92 ± 0.01
	Leaf Curl	0.90 ± 0.02	0.92 ± 0.01	0.91 ± 0.01	
	Leaf Spot	0.98 ± 0.00	0.92 ± 0.02	0.95 ± 0.01	
	Whitefly	0.89 ± 0.02	0.92 ± 0.01	0.91 ± 0.01	
	Yellowish	0.90 ± 0.01	0.92 ± 0.01	0.91 ± 0.01	
EfficientNetB0 + Attention	Healthy	0.97 ± 0.01	0.98 ± 0.01	0.98 ± 0.01	0.98 ± 0.00
	Leaf Curl	0.99 ± 0.00	0.98 ± 0.01	0.98 ± 0.01	
	Leaf Spot	0.99 ± 0.00	0.98 ± 0.01	0.98 ± 0.01	
	Whitefly	0.97 ± 0.01	0.98 ± 0.00	0.98 ± 0.01	
	Yellowish	0.98 ± 0.01	0.98 ± 0.01	0.98 ± 0.01	
VGG‐EffAttnNet (Proposed Model)	Healthy	0.99 ± 0.00	0.99 ± 0.00	0.99 ± 0.00	0.99 ± 0.00
	Leaf Curl	1.00 ± 0.00	0.99 ± 0.00	0.99 ± 0.00	
	Leaf Spot	0.98 ± 0.01	0.99 ± 0.00	0.99 ± 0.00	
	Whitefly	0.99 ± 0.00	0.99 ± 0.00	0.99 ± 0.00	
	Yellowish	0.99 ± 0.00	0.99 ± 0.00	0.99 ± 0.00	

## 8 State‐of‐the‐Art Comparison

Table 7 summarize the State‐of‐the‐Art comparison that analyzes numerous deep learning approaches used for chili plant disease classification. Hamim et al. (Gulzar and Ünal 2025) used MobileNet on a dataset of 300 images to achieve a level of 97.18%, proving the efficacy of the model in lightweight classifying tasks. Srinivasulu et al. (Gulzar et al. 2024) applied RNDDNet to a more considerable dataset of 3800 images to marginally advance performance to 98.09%, suggesting increased generalization powers. Murint et al. (Hamim and Jony 2024) implemented VGG16 on 250 images and achieved a 94% accuracy, testifying to its performance even with a fairly smaller dataset. Li et al. (Srinivasulu and Maiti 2024) introduced MCSAM, which reached 91.2% accuracy on 500 images and proved to be reliable but improvable. As a reference, the model with VGG‐EffAttnNet on a much bigger dataset of 5000 images is more accurate than any other method, achieving an astonishing accuracy of 99%. Utilizing attention mechanisms and the hybridization of VGG16 and EfficientNet enhances the model's ability to focus on discriminative features, leading to improved classification results. These results validate the superiority of hybrid deep learning approaches to enhancing chili plant disease detection over existing methods in accuracy and scalability. Although the performance improvement of the proposed model over InceptionV3 (99% vs. 98.83%) appears modest, it is consistent across multiple runs and critical in high‐stakes agricultural applications where small gains can reduce misdiagnosis. The standard deviations reported through cross‐validation indicate stable and reliable performance.

**TABLE 7:** State‐of‐the‐art comparison.

References	Dataset	Techniques	No. of images	Results
Gulzar and Ünal (2025)	Chili leaf Disease	MobileNet	300	Accuracy: −97.18%
Gulzar et al. (2024)	Chili plant disease	RNDDNet	3800	Accuracy: −98.09%
Hamim and Jony (2024)	Chili plant disease	VGG16	250	Accuracy: −94%
Srinivasulu and Maiti (2024)	Chili plant Disease	MCSAM	500	Accuracy: −91.2%
Proposed model	Chili plant disease	VGG‐EffAttnNet	5000	Accuracy: −99%

## 9 Conclusions and Future Work

The study presents a hybrid deep learning model integrating VGG16 and EfficientNetB0 with attention mechanisms and Monte Carlo Dropout (MCD) for accurate and automated classification of chili plant diseases. The proposed model effectively leverages hierarchical feature learning from VGG16 and computational efficiency from EfficientNetB0, while the attention mechanism enhances disease‐relevant feature extraction. Experimental results demonstrate the superior performance of the hybrid model, achieving 99% accuracy, surpassing individual models (VGG16: 96.8%, EfficientNetB0: 96.5%), with an F1‐score of 99% across all disease categories. The study underscores the potential of deep learning‐based automated detection for early disease identification, precision agriculture, and sustainable farming practices. Future work aims to deploy the model through real‐world field trials and collect data from diverse agro‐climatic regions to enhance its robustness and generalizability under practical agricultural conditions. Additionally, deploying the model on mobile devices presents challenges such as increased model complexity, higher inference latency on low‐power processors, and energy constraints. These will be addressed through model optimization techniques like pruning, quantization, and knowledge distillation. In future work, the plan is to incorporate explainable AI (XAI) techniques such as Grad‐CAM to visualize which parts of the leaf image contribute most to the classification decision. Incorporating explainable AI will be vital for building user trust and supporting practical decision‐making in real‐world agricultural applications. This will help bridge the gap between high model accuracy and practical usability, enabling users, especially farmers and agronomists, to better understand and trust the model's outputs. Although the dataset was expanded to 5000 images through augmentation, it may still lack the full diversity found in real‐world field conditions. Future work will include the collection of field images under varying environmental settings to further enhance generalizability and reduce the dataset's current homogeneity. Additionally, federated learning will be explored for decentralized disease diagnostics, ensuring scalability and accessibility across diverse agricultural landscapes. To further enhance efficiency, future research will also focus on automated architecture search (AutoML), model compression, and multimodal data fusion (e.g., combining image data with sensor/environmental inputs) to reduce resource usage and improve contextual prediction. Exploring multimodal data fusion by integrating image data with environmental and sensor inputs will enhance robustness and contextual accuracy. Enhancing interpretability will also support actionable decision‐making in agricultural scenarios where expert guidance may be limited. The research paves the way for next‐generation AI‐driven plant disease detection systems, offering cost‐effective, scalable, and efficient solutions for modern agriculture.

## Author Contributions

**Ritu Rani:** conceptualization (equal), formal analysis (equal), investigation (equal), software (equal), writing – original draft (equal). **Salil Bharany:** conceptualization (equal), methodology (equal), validation (equal), writing – review and editing (equal). **Dalia H. Elkamchouchi:** data curation (equal), formal analysis (equal), software (equal). **Ateeq Ur Rehman:** conceptualization (equal), methodology (equal), project administration (equal), writing – review and editing (equal). **Rahul Singh:** formal analysis (equal), investigation (equal), methodology (equal), visualization (equal). **Seada Hussen:** project administration (equal), validation (equal), writing – review and editing (equal).

## Ethics Statement

The authors have nothing to report.

## Consent

The authors have nothing to report.

## Conflicts of Interest

The authors declare no conflicts of interest.

## Data Availability

The dataset used in this study is publicly available on Kaggle: https://www.kaggle.com/datasets/dhenyd/chili‐plant‐disease.
